# Unraveling the relationship between high-sensitivity C-reactive protein and frailty: evidence from longitudinal cohort study and genetic analysis

**DOI:** 10.1186/s12877-024-04836-2

**Published:** 2024-03-04

**Authors:** Yu-Feng Luo, Zi-Jian Cheng, Yan-Fei Wang, Xi-Yuan Jiang, Shu-Feng Lei, Fei-Yan Deng, Wen-Yan Ren, Long-Fei Wu

**Affiliations:** 1 Center for Genetic Epidemiology and Genomics, School of Public Health, Medical College of Soochow University, Suzhou, Jiangsu People’s Republic of China; 2 Jiangsu Key Laboratory of Preventive and Translational Medicine for Geriatric Diseases, Soochow University, Suzhou, Jiangsu People’s Republic of China; 3 MOE Key Laboratory of Geriatric Diseases and Immunology, Soochow University, Suzhou, Jiangsu People’s Republic of China; 4 Center of Osteoporosis, Kunshan Hospital of Traditional Chinese Medicine, Kunshan, Jiangsu People’s Republic of China; 5 Cambridge-Suda Genomic Resource Center, Jiangsu Key Laboratory of Neuropsychiatric Diseases, Medical College of Soochow University, Suzhou, Jiangsu People’s Republic of China

**Keywords:** Hs-CRP, Frailty, Cohort study, Genetics, GWAS

## Abstract

**Background:**

This study aimed to investigate the association of high-sensitivity C-reactive protein (hs-CRP) with incident frailty as well as its effects on pre-frailty progression and regression among middle-aged and older adults.

**Methods:**

Based on the frailty index (FI) calculated with 41 items, 6890 eligible participants without frailty at baseline from China Health and Retirement Longitudinal Study (CHARLS) were categorized into health, pre-frailty, and frailty groups. Logistic regression models were used to estimate the longitudinal association between baseline hs-CRP and incident frailty. Furthermore, a series of genetic approaches were conducted to confirm the causal relationship between CRP and frailty, including Linkage disequilibrium score regression (LDSC), pleiotropic analysis, and Mendelian randomization (MR). Finally, we evaluated the association of hs-CRP with pre-frailty progression and regression.

**Results:**

The risk of developing frailty was 1.18 times (95% CI: 1.03–1.34) higher in participants with high levels of hs-CRP at baseline than low levels of hs-CRP participants during the 3-year follow-up. MR analysis suggested that genetically determined hs-CRP was potentially positively associated with the risk of frailty (OR: 1.06, 95% CI: 1.03–1.08). Among 5241 participants with pre-frailty at baseline, we found pre-frailty participants with high levels of hs-CRP exhibit increased odds of progression to frailty (OR: 1.39, 95% CI: 1.09–1.79) and decreased odds of regression to health (OR: 0.84, 95% CI: 0.72–0.98) when compared with participants with low levels of hs-CRP.

**Conclusions:**

Our results suggest that reducing systemic inflammation is significant for developing strategies for frailty prevention and pre-frailty reversion in the middle-aged and elderly population.

**Supplementary Information:**

The online version contains supplementary material available at 10.1186/s12877-024-04836-2.

## Introduction

Frailty is characterized by decreased physiological reserve, reduced resilience, and increased vulnerability to stressors. Older adults with frailty are often associated with various adverse outcomes such as falls, fractures, disability, hospitalization, and mortality [1]. The prevalence of frailty is estimated to be 18% among community-dwelling older people aged 60 years and over worldwide [2]. However, frailty is a dynamic process that may be delayed or reversed. Therefore, identifying modifiable risk factors for frailty is critical for developing strategies to prevent progression and promote pre-frailty regression [3–5].

Frailty is characterized by multi-system dysregulation, the pathophysiology of which is not clearly understood. However, emerging evidence suggests chronic, low-grade inflammation is closely related to frailty. Many studies identified several SNPs in genes related to inflammatory pathways that may increase the risk of developing frailty in older adults [6–8]. Some researchers suggested a genetic basis, with heritability estimates between 30 and 45% [9–11]. Candidate gene association studies for frailty have suggested the involvement of genes in inflammatory pathways, including IL-18 [12]. Previous population-based cohort studies also highlighted the importance of elevated inflammation in frailty incidents. For example, a direct association between frailty and high inflammation factors, as marked by IL-6 and CRP, has been observed [13]. Similar results were obtained regarding white blood cells and fibrinogen levels. The mechanisms underlying the effects of inflammation on frailty are likely multifactorial. Chronic inflammation may contribute to the development of frailty through various mechanisms, including increased oxidative stress, muscle wasting, reduced immune function, and reduced mobility [14].

As a routinely measured parameter of systemic inflammation, the relationship of CRP in frailty has been examined, but the findings were inconsistent. Through observational studies, elevated serum CRP levels have been recognized as a risk factor for frail individuals. However, some longitudinal studies did not observe an association between higher CRP and increased risk of frailty [15]. Interestingly, another study reported that more elevated CRP was independently associated with an increased risk of frailty only in women, suggesting the effects of CRP on frailty varies by gender [13]. Such discrepancies may be due to potential confounders unmeasured that might affect their relationship. Of note, epidemiologic associations are vulnerable to reverse causation biases because frailty may lead to dysregulation of CRP levels. What are the causal roles of CRP on frailty? In addition, previous observational studies mainly examined the relationship between inflammatory factors CRP and the risk of frailty. However, the frailty state (non-frailty, pre-frailty, and frailty) is dynamic. Whether CRP plays any role in pre-frailty progression or regression?

To address the above questions, we first replicated previous observational findings by examining the association of CRP and frailty among Chinese middle-aged and older adults based on the China Health and Retirement Longitudinal Study (CHARLS). Then, we systematically evaluated the complex relationship between CRP and frailty from the perspective of shared genetics, pleiotropy, and causality. First, the genetic correlation between CRP with estimated frailty was assessed. We then applied a novel method PLACO to identify genes contributing to pleiotropy. Moreover, using genetic variants as instrumental variables, an MR study was conducted to infer the possible causal relationship between exposure (CRP level) and outcome (frailty). Finally, Logistic regression analysis was applied to assess the association of CRP with pre-frailty progression or regression.

## Methods

### Study population

**Fig. 1:**
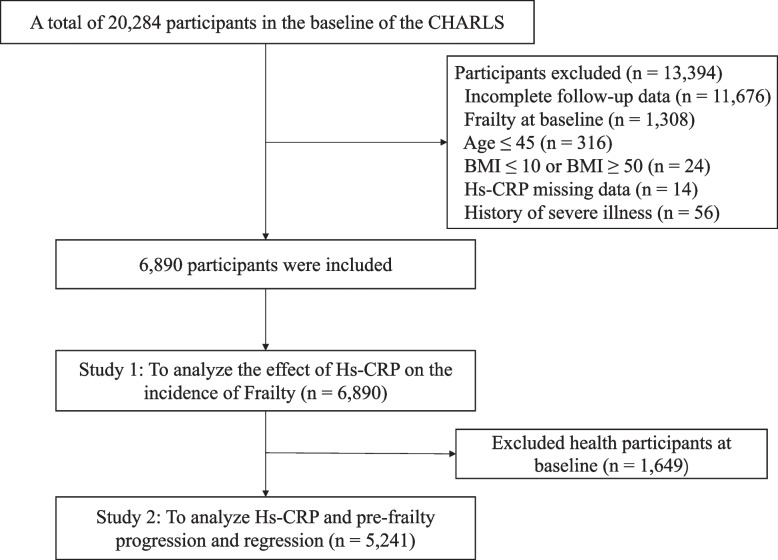
Flowchart of participants selection from the China Health and Retirement Longitudinal Study (CHARLS)

The China Health and Retirement Longitudinal Study (CHARLS) is a long-term study of middle-aged and older people from all over China. Eligible people are chosen through multiple rounds of random sampling [16] from 150 counties of 28 provinces [17]. The sampling method and questionnaire of the CHARLS have been described elsewhere [18]. The baseline survey of CHARLS was fielded from June 2011 to 2012. Respondents were followed up every 2 years with physical measurements and fasting blood samples collected. Face-to-face, computer-assisted personal interviews were used to collect information. In this study, data from the 2015 (baseline) and 2018 waves were used for analysis. We selected people aged 45 years or older, excluded 1308 people who were already frail in 2015, and excluded people with a history of disease and a BMI of 10 or > 50. Finally, 6890 participants were included in the following analysis (Fig. 1). All participants provided written informed consent. The study was approved by Peking University’s Ethics Review Board (IRB00001052–11015) [19].

### Assessment of hs-CRP and data collection

Venous blood samples were collected by trained staff at the local Chinese Center for Disease Control and Prevention. Plasma samples were collected and stored in 0.5-ml cryovials at − 80 °C for testing at the Youmen Clinical Laboratory Centre of Capital Medical University. Fasting blood samples were collected at each wave to measure hs-CRP, HbA1c, triglycerides (TG), and high-density lipoprotein cholesterol. The reference values (OR = 1) were set at hs-CRP = 1.46. A nonlinear association was found between the hs-CRP and frailty risk (P for non-linearity = 0.005, Fig. S1), and the curve was approximately inverted L-shaped. Due to the non-normal distribution of hs-CRP, the data were categorized into tertiles: low-level hs-CRP (Tertile 1, < 0.90 mg/L), medium-level hs-CRP (Tertile 2, 0.90–2.00 mg/L), and high-level hs-CRP (Tertile 3, > 2.00 mg/L). Information was collected on demographic factors (including age and sex), health behaviors (including smoking and alcohol consumption), and 14 medical histories. Anthropometric parameters, including weight, height, and waist circumference (WC), were measured according to a standard protocol. BMI was calculated by dividing an individual’s weight by the square of their height (kg/m2) [20].

### Ascertainment of pre-frailty, frailty, and health

Forty-one indicators were collected from 2015 and 2018 to calculate the frailty index (FI). FI is a continuous variable with values ranging from 0.00 to 1.00, with higher values indicating a poorer and more vulnerable status [21]. Based on the 2015 and 2018 FI, the mean FI score for each respondent over the study period was calculated and classified as health (< 0.10), pre-frail (0.10–0.25), and frailty (≥0.25). The FI for each respondent was calculated by dividing the number of impairments a person had by the total number of impairments (Table S1). In line with previous literature, we did not assign weights to individual indicators that were interrelated. Incomplete data were imputed with the multiple imputation method by chaining two equations [21].

### Statistical analysis

The data were described using means (standard deviation) for continuous variables and frequencies (%) for categorical variables. The student’s *t*-test and Mann-Whitney U test were used to analyse continuous variables, and the chi-square test was used to analyse categorical variables. A one-way ANOVA was used to compare characteristics between different hs-CRP groups. Based on the follow-up results, participants were divided into three groups: (i) healthy, (ii) pre-frailty, and (iii) frailty. Multiple logistic regression analyses were performed to obtain the association between hs-CRP and frailty in the tertile. The ratios (ORs) and 95% confidence intervals (CIs) between pre-frailty in 2015 and progressing or returning to normal in 2018 were used. Three different models are presented: Model 1, which requires no adjustment; Model 2, which adjusts for sex and age; and Model 3, which adjusts for age, sex, body mass index, marital state, smoke, systolic blood pressure, diastolic blood pressure, drinking status, sleep duration, hypertension, hyperlipidemia, diabetes, cardiovascular disease. Subgroup analyses by gender grouping were conducted using Model 1, Model 2, and Model 3, which adjusts for sex, age, body mass index, marital status, smoking, systolic blood pressure, diastolic blood pressure, drinking status, sleep duration, hypertension, hyperlipidemia, diabetes, cardiovascular disease. Stratified analyses according to sex, age, BMI, the presence of hypertension, the disease history of diabetes, and the disease history of cardiovascular disease and dyslipidemia were conducted mainly using Model 3 and the interaction of these variables with hs-CRP. Sensitivity analyses were performed to verify whether the absence of relevant covariates affected the results.

Linkage disequilibrium score regression (LDSC) was utilized to estimate heritability and assess the genetic correlation between CRP and frailty using summary-level genome-wide association study (GWAS) data [22]. The genetic correlation represents the degree of shared genetic basis between CRP and frailty, indicating whether they have overlapping underlying genetic factors. According to the manual LDSC, the GWAS summary statistics data were first reformatted into the LDSC format using the default parameter. LDSC performs a regression analysis by regressing the summary-level association statistics (typically z-scores) from GWAS studies against the LD scores. This regression allows for estimating heritability, genetic correlation, and genetic covariance between traits. Because most GWAS samples were primarily from European ancestry, we used the LD scores of 1000 Genomes European data as LD reference [23]. The Bonferroni method was used to correct the *P* value of LDSC. A total of 14 tests were conducted. Therefore, the significant threshold was *P* < 3.6 × 10^−3^ (0.05/14).

Pleiotropic analysis under composite null hypothesis (PLACO) is a novel method that could be utilized to understand the genetic basis of complex traits by identifying genetic variants that affect CRP and frailty simultaneously [24]. We calculated the squares of Z scores for each variant and removed the SNPs with extreme Z2 (> 80). Also, we estimated the correlation matrix of Z, considering the potential correlation between CRP and frailty. Then, a level-α intersection–union test (IUT) method was used to test the hypothesis of no pleiotropy: The null hypothesis H_0_ is H_0_: H_00_∪H_01_∪H_02_ and alternative hypothesis. H_1_ could be further expressed as:

$$\mathrm H1:H_{00}^a\cap H_{01}^a\cap H_{02}^a,$$

(where)

$$\mathrm H00:\beta_{CRP}=\beta_{Frailty}=0,$$

$$\mathrm H01:\beta_{CRP}=0,\beta_{Frailty}\neq0$$

(and)

$$\mathrm H02:\beta_{CRP}\neq0,\beta_{Frailty}=0.$$

The H^a^ represents the complement of H. β_CRP_ and β_Frailty_ represents effect size of CRP and frailty, respectively. Then, the final *P*-values for IUT test are the maximum of P-values for testing H_0_ versus H_1_, which could be instead by an asymptotic approximation:

1 $${\displaystyle \begin{array}{l}{\hat{\textrm{p}}}_{{\textrm{z}}_{\textrm{CRP}} {\textrm{z}}_{Frailty}}=F\left({\textrm{z}}_{\textrm{CRP}}{\textrm{z}}_{Frailty}/\sqrt{Var\left({\textrm{z}}_{CRP}\right)}\right)\\ {}+F\left({\textrm{z}}_{\textrm{CRP}}{\textrm{z}}_{Frailty}/\sqrt{Var\left({\textrm{z}}_{F\textrm{railt}y}\right)}\right)-F\left({\textrm{z}}_{\textrm{CRP}}{\textrm{z}}_{Frailty}\right)\end{array}}$$

In Eq. (1), Z_CRP_ represents single-trait test statistics for CRP, Z_Frailty_ represents single-trait test statistics for Frailty; F(x) represents the two-sided tail probability of a normal product distribution for x; Var(x) is the expectation of the squared deviation of x from its mean.

The data of C-reactive protein (hs-CRP) phenotypes were obtained from the IEU Open GWAS database (https://gwas.mrcieu.ac.uk/), a database containing 42,335 GWAS summary datasets. The GWAS summary data for frailty was obtained from the IEU Open GWAS database, which performed a GWAS of a frailty index in European descent UK Biobank participants (*n* = 164,610) and Swedish Twin Gene participants (*n* = 10,616). FI calculation was based on 49 or 44 self-reported items on symptoms, disabilities, and diagnosed diseases for UK Biobank and Twin Gene [25]. The information about the effect allele, effect size (beta or odds ratio), standard error, and *P* value were obtained from the GWAS studies.

To establish a causal effect of hs-CRP traits on frailty. The MR method needs to satisfy the following three assumptions: (i) the genetic variant (Instrumental variable) is robustly associated with hs-CRP (Exposure); (ii) the genetic variant does not share common causes (potential confounding factors) with frailty (Outcome); and (iii) the genetic variant affects frailty (Outcome) exclusively through its effect on hs-CRP traits (Exposure).

Five main methods are used to estimate causal effects: the random effects inverse variance weighted (IVW) method was utilized in the primary MR analyses. Furthermore, MR-Egger regression, the weighted median, simple mode, and weighted mode were performed as complementary analyses. We utilized the IVW method as the primary analysis for its efficiency in estimating the causal effect. The weighted median was used as an auxiliary method when the heterogeneity was significant, and the MR-Egger regression method was used to assess the pleiotropy by intercept test. Several sensitivity analyses were used to correct the causal estimates. Heterogeneity between SNPs included in each analysis was first tested using the Cochran Q test, and if heterogeneity existed, then random effects IVW was used. A combined sensitivity analysis was then performed to verify the robustness of our results. The intercept of the MR-egger method was used to test for horizontal multiplicity, and the MR multiplicity residual sum and outlier method (MR-PRESSO) was used to detect potential outliers [26]. The MR pleiotropy residual sum and outlier (MR-PRESSO) method was capable of identifying outlying SNPs and providing reliable estimates with outlier correction [27]. A leave-one-out analysis was performed to evaluate the stability of these genetic variants on CRP traits. In order to increase the reliability of the results, we added three tests, namely Maximum likelihood, penalized, IVW and MR-RAPS. The maximum likelihood method enabled us to make a valid estimation in the case of measurement error in SNP-exposure association [28], and the penalized IVW method could penalize the SNPs with pleiotropy [29]. The MR total adjusted profile score (MR-RAPS) method was robust to the violations of key MR assumptions [30].

## Results

### Baseline characteristics

A total of 6,890 participants with a mean age of 60.39 ± 9.20 years were included in this study. According to the frailty index (FI) calculated with 41 measured items, participants were divided into a health group, a pre-frailty group, and a frailty group. After 3 years of follow-up, 2669 individuals in health, 3700 in pre-frailty, and 521 in frailty were observed with their baseline characteristics shown in Table 1. We found that the age, sex, drinking, smoking, marital status, ABSI, LDL-c, UA, HbA1c, SBP, and health groups significantly differed from the pre-frailty and frailty groups (*P* <  0.05)**.** Moreover, the trend test showed a dose-response association between clinical indicators and frailty increase except for BMI, HDL-c, UA, and DBP (*P*_trend_ <  0.05).

**Table 1:** Baseline characteristics of participants stratified by frailty level(*n* = 6890)

	Total	Health (Tertile1 < 0.1)	Pre-frailty (Tertile3 0.1–0.25)	Frailty (Tertile2 ≥ 0.25)	*P* value	*P* for trend
Sample size (n)	6890	2669	3700	521		
hs-CRP (mg/l)	2.37 ± 4.48	2.32 ± 4.55	2.34 ± 4.44	2.80 ± 4.41	0.12	0.03
Age (years)	60.39 ± 9.20	57.96 ± 8.32	61.35 ± 9.15^*^	66.16 ± 10.05^*^	< 0.01	< 0.01
Marital status (%)					< 0.01	< 0.01
Married and living with a spouse	5875 (85.27%)	2360 (88.42%)	3115 (84.19%) ^*^	400 (76.78%) ^*^		
Married but living without a spouse	323 (4.69%)	125 (4.68%)	182 (4.92%) ^*^	16 (3.07%) ^*^		
Single, divorced, and Windowed	692 (10.04%)	184 (6.89%)	403 (10.89%) ^*^	105 (20.15%) ^*^		
Female (%)	3599 (51.66%)	1166 (43.69%)	2067 (55.87%) ^*^	326 (62.57%) ^*^	< 0.01	< 0.01
Smoking (%)	2585 (37.52%)	1176 (44.06%)	1278 (34.54%) ^*^	131 (25.14%) ^*^	< 0.01	< 0.01
Drinking (%)	467 (6.78%)	243 (9.11%)	207 (5.59%) ^*^	17 (3.26%) ^*^	< 0.01	< 0.01
BMI (kg/m^2^)	24.08 ± 3.59	24.07 ± 3.45	24.07 ± 3.61	24.20 ± 4.10	0.61	0.46
ABSI	8.24 ± 0.65	8.19 ± 0.63	8.25 ± 0.65^*^	8.40 ± 0.70^*^	< 0.01	< 0.01
TG (mg/dl)	143.85 ± 90.63	140.89 ± 90.55	145.16 ± 90.51	149.65 ± 91.53	0.02	0.04
HDL-c (mg/dl)	50.89 ± 11.35	50.68 ± 11.13	50.96 ± 11.46	51.28 ± 11.70	0.20	0.27
LDL-c (mg/dl)	101.84 ± 28.46	100.70 ± 28.07	102.19 ± 28.27	105.16 ± 31.36^*^	< 0.01	< 0.01
UA (mg/dl)	4.90 ± 1.38	4.98 ± 1.39	4.85 ± 1.36^*^	4.90 ± 1.37	< 0.01	0.19
HbA1c (%)	5.95 ± 0.94	5.84 ± 0.78	5.99 ± 0.96^*^	6.24 ± 1.39^*^	< 0.01	< 0.01
SBP (mmHg)	127.75 ± 19.02	125.45 ± 17.81	128.38 ± 19.27^*^	135.084 ± 20.938^*^	< 0.01	< 0.01
DBP (mmHg)	75.97 ± 11.65	75.86 ± 11.44	75.93 ± 11.85	76.77 ± 11.24^*^	0.24	0.11

*BMI* Body mass index, *ABSI* A Body Shape Index, *TG* Triglycerides, *HDL-c* High-density lipoprotein-cholesterol, *LDL-c* Low-density lipoprotein-cholesterol, *UA* Uric acid, *HbA1c* Haemoglobin A1c, *hs-CRP* High-sensitivity C-reactive protein, *SBP* Systolic blood pressure, *DBP* Diastolic blood pressure, *TC* Triglycerides

They were compared using one-way analysis of variance or χ ^2^ test, as appropriate, with Bonferroni’s correction for multiple comparisons

*P<0.05, compared with remained as health

### Observation study for the association between hs-CRP and frailty

**Fig. 2:**
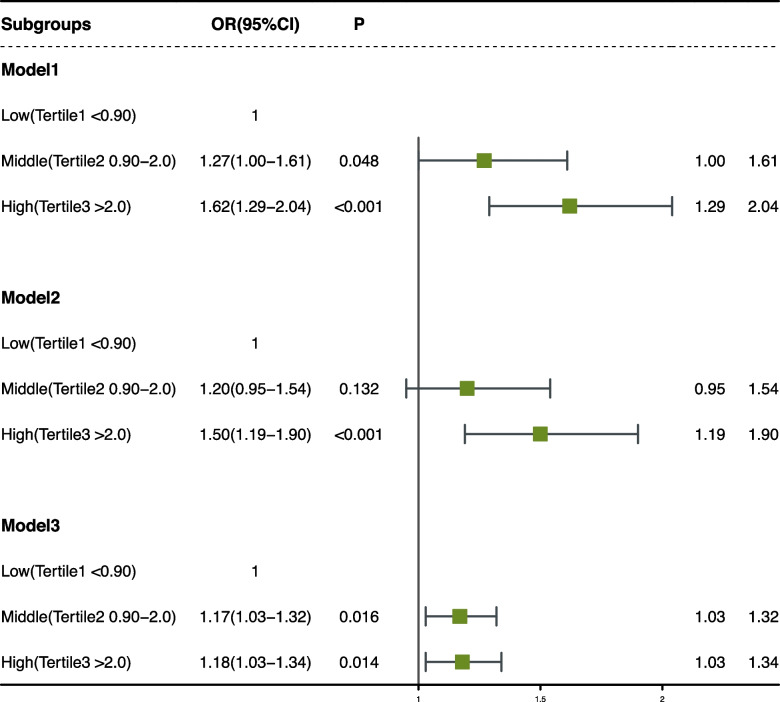
Forest plot of logistic regression analysis of Hs-CRP and frailty; model 1: unadjusted; model 2: adjusted for age, sex; model 3: adjusted for age, sex, body mass index, marital state, smoke, systolic blood pressure, diastolic blood pressure, drinking status, sleep duration, hypertension, hyperlipidemia, diabetes, cardiovascular disease

The association of hs-CRP and frailty is shown in Fig. 2. In the unadjusted model (Model 1), participants with both moderate (OR:1.27, 95% CI: 1.00–1.61) and high (OR:1.62, 95% CI: 1.29–2.04) level hs-CRP had increased odds of frailty when compared with participants with low-level hs-CRP. For participants with a high level of hs-CRP, the association of hs-CRP with the odds of frailty remains significant after controlling for multivariable (OR:1.50, 95% CI 1.19–1.90 in Model 2 and OR:1.18, 95% CI: 1.03–1.34 in Model 3). Sensitivity analyses upon the exclusion of participants who had no age, drink, smoke, or BMI data, the risk of frailty was still higher in participants with high levels of hs-CRP than those with low levels of hs-CRP in both unadjusted model and multivariable-adjusted model (Fig. S2). We further performed stratified analyses to examine the association of tertiles of hs-CRP with frailty. Similar trends were obtained in that the higher the hs-CRP levels in participants with health, the greater their risk of progression to frailty. However, several associations were no longer evident or statistically significant (Table 2).

**Table 2:** Association between the Hs-CRP levels and Frailty in subgroups

	N	Low (Tertile1 < 0.90) OR (95%CI)	Middle (Tertile2 0.90–2.0) OR (95%CI)	High (Tertile3 > 2.0) OR (95%CI)	*P* for interaction
Age,years					0.586
< 60	3308	1 (Reference)	1.40(1.18–1.66)	1.29(1.07–1.55)	
≥60	3582	1 (Reference)	0.95(0.79–1.14)	1.07(0.89–1.29)	
Sex					0.093
Male	3331	1 (Reference)	1.13(0.95–1.35)	1.06(0.88–1.27)	
Female	3559	1 (Reference)	1.18(0.99–1.41)	1.31(1.09–1.58)	
BMI,kg/m^2^					0.137
< 24	3526	1 (Reference)	1.06(0.90–1.25)	1.11(0.92–1.33)	
24–27.9	2373	1 (Reference)	1.32(1.06–1.65)	1.30(1.04–1.63)	
≥28	952	1 (Reference)	1.54(0.98–2.42)	1.50(0.97–2.32)	
The presence of hypertension					0.645
Yes	1476	1 (Reference)	1.31(0.96–1.79)	1.18(0.87–1.60)	
No	5414	1 (Reference)	1.13(0.99–1.30)	1.18(1.02–1.36)	
The presence of hyperlipidemia					0.386
Yes	675	1 (Reference)	1.24(0.78–1.99)	1.32(0.83–1.99)	
No	6215	1 (Reference)	1.16(1.02–1.32)	1.15(1.01–1.32)	
The disease history of diabetes					0.392
Yes	358	1 (Reference)	1.91(0.98–3.76)	0.88(0.46–1.65)	
No	6532	1 (Reference)	1.14(1.01–1.30)	119(1.04–1.36)	
The disease history of cardiovascular disease					0.733
Yes	778	1 (Reference)	1.29(0.84–1.98)	1.07(0.70–1.62)	
No	6112	1 (Reference)	1.15(1.01–1.31)	1.19(1.04–1.37)	

All analyses adjusted for age, sex, body mass index, marital state, smoke, systolic blood pressure, diastolic blood pressure, drinking status, sleep duration, hypertension, hyperlipidemia, diabetes, cardiovascular disease

### Shared genetic and pleiotropic correlation between CRP and frailty

**Fig. 3:**
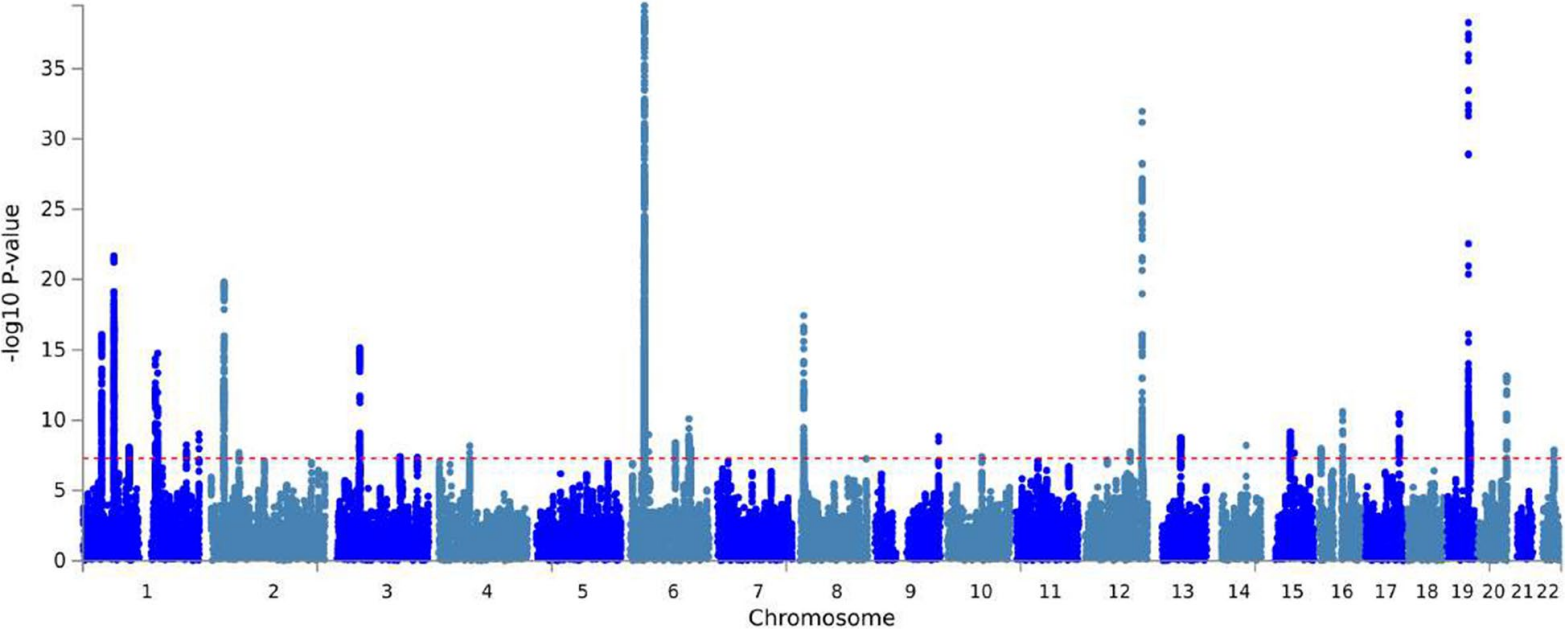
Manhattan plot of the PLACO results. Horizontal line represents the significance of 5E-8. r^2^ threshold to define independent significant SNPs was set to 0.2 and maximum distance between LD blocks to merge into a locus was set to 500 kb

Then, linkage disequilibrium score regression (LDSC) was constructed to estimate single-nucleotide polymorphisms (SNP)-based heritability and assess the genetic correlation between CRP and frailty. The SNP-based heritability was estimated to be 4.42% (SE = 0.003) for CRP and 10.94% (SE = 0.005) for frailty with LDSC. Then, a negative genetic correlation between CRP and frailty was observed (r_g_ = − 0.39, *P* = 9.96E-22), suggesting a potential shared genetic mechanism between CRP and frailty. Furthermore, PLACO analysis was performed to investigate the specific genetic loci shared by CRP and frailty. A total of 51 pleiotropic lead SNPs that were associated with both CRP and Frailty (*P* < 5.00E-08) mapping to 34 genomic risk loci were identified (Fig. 3).

### Two-sample MR analysis

**Fig. 4:**
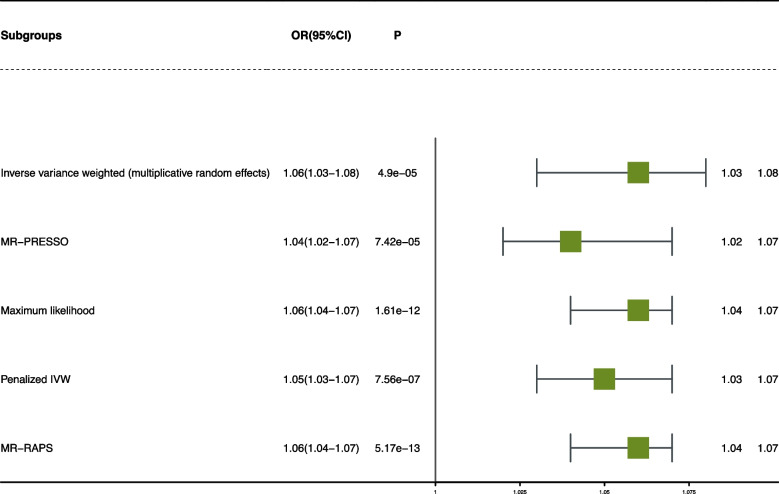
Forest plot of Mendelian randomization for the relationship between Hs-CRP and frailty. The random effects inverse variance weighted (IVW) method was utilized in the main MR analyses. The MR pleiotropy residual sum and outlier (MR-PRESSO) method, the maximum likelihood method and the MR robust adjusted profile score (MR-RAPS) method were performed as complementary analyse

To further explore whether there is a causal relationship between hs-CRP and frailty, MR analysis was conducted by using genetic variants as instrumental variables. We found a modest association between hs-CRP and frailty in the main inverse-variance weighted (OR:1.06, 95% CI: 1.03 to 1.08, *P* = 4.9E-05) (Fig. 4). A positive causal association between hs-CRP and frailty was also observed for Maximum likelihood analysis, Penalized IVW analysis, and MR-RAPS analysis. The scatter plot and funnel plot are shown in (Figs. S3 and S4). For instrumental variable (IV) selection, 348 single-nucleotide polymorphisms (SNPs) that have a robust association with hs-CRP at the threshold of statistical significance (*P* < 5.00E-08) were selected. To remove bias from linkage disequilibrium (LD), a clumping process was conducted with the European population and LD between SNPs (R^2^ <  0.01, kb = 5000). Finally, leaving 297 SNPs as IVs for further analysis (Table S2).

The instrumental variable was selected only from the European population. In addition, we removed six SNPs with intermediate allele frequency palindromes. As a result, estimates for 293 SNPs were included in the analysis of hs-CRP and frailty. For sensitivity analysis, Cochran’s Q test showed that there is significant heterogeneity for the causal effect of hs-CRP on frailty (*Q-pval* <  0.001), then Inverse variance weighted (multiplicative random effects) was conducted. The MR-Egger intercept test demonstrated that our results were influenced by pleiotropy (*P* <  0.001). MR-PRESSO analysis showed horizontal pleiotropy (*P*_*MR-PRESSO*_ <  0.001). Lastly, the leave-one-out sensitivity analysis confirmed the stability of the causal inference (Fig. S5).

### Association of hs-CRP with prefrailty progression and regression

Moreover, we examined the relationship between hs-CRP and the progression or regression of pre-frailty (Table 3). Pre-frailty participants with high levels of hs-CRP had increased odds of progression to frailty (OR:1.66, 95% CI: 1.31–2.11) compared with participants with low levels of hs-CRP. Conversely, participants with high levels of hs-CRP had reduced odds of regression to health (OR:0.76, 95% CI: 0.66–0.88) in the unadjusted model (Model 1). Similarly, we found participants with high hs-CRP have increased odds of progression to frailty (OR:1.54, 95% CI: 1.21–1.96, model 2 and OR:1.39, 95% CI: 1.09–1.79, Model 3) and decreased odds of regression to health (OR:0.79, 95% CI: 0.68–0.91, model 2 and (OR:0.84, 95% CI: 0.72–0.98, Model 3) in the multivariable-adjusted model, compared with participants with the low level of hs-CRP.

**Table 3:** Hs-CRP and Pre-frailty regression and progression

No. of cases/total	Model1^a^	Model2^b^	Model3^c^	
	OR (95%CI)	OR (95%CI)	OR (95%CI)	
Frailty progression				
Low (Tertile1 <0.90)	118/1602	1	1	1
Middle (Tertile2 0.90–2.0)	170/1838	1.28 (1.00–1.64)	1.23 (0.96–1.58)	1.19 (0.92–1.54)
High (Tertile3 >2.0)	210/1801	1.66 (1.31–2.11)	1.54 (1.21–1.96)	1.39 (1.09–1.79)
* P* for trend		< 0.001	< 0.001	< 0.001
Frailty regression				
Low (Tertile1 <0.90)	543/1602	1	1	1
Middle (Tertile2 0.90–2.0)	581/1838	0.90 (0.78–1.04)	0.91 (0.79–1.053)	0.93(0.80–1.08)
High (Tertile3 >2.0)	505/1801	0.76 (0.66–0.88)	0.79 (0.68–0.91)	0.84(0.72–0.98)
*P* for trend		0.001	< 0.001	< 0.001

^a^Unadjusted

^b^Adjusted for age, sex

^c^Adjusted for age, sex, body mass index, marital state, smoke, systolic blood pressure, diastolic blood pressure, drinking status, sleep duration, hypertension, hyperlipidemia, diabetes, cardiovascular disease

## Discussion

In this study, we replicated previous studies to estimate the effect of inflammatory factors, as assessed by hs-CRP, on the risk of frailty in a large Chinese middle-aged and elderly cohort. Our results suggested that higher levels of hs-CRP were associated with a higher risk of frailty. This is also the first time to systematically combine a series of genetic approaches, such as LDSC, pleiotropic analysis, and MR methods, to evaluate the relationship between CRP and frailty. Of note, this study also first found that higher levels of hs-CRP are associated with increased odds of progression. In comparison, lower levels of hs-CRP are associated with increased regression odds in pre-frailty participants.

Previous observational studies have examined the relationship between chronic low-level systemic inflammation and frailty but yielded contradictory results. CRP is a routinely measured biomarker of inflammation associated with many chronic diseases. A review explored the correlation between elevated serum hs-CRP levels and increased incidence of frailty in older adults through 29 studies from 2004 to 2016 involving ethnically diverse populations [31]. Recently, Cheng et al. found that higher CRP and WBC accelerated the progression of frailty, particularly in younger groups. Meanwhile, studies on CHS cohorts have found higher levels of inflammatory factors such as hs-CRP and IL-6 at baseline in those who present with frailty compared to those who do not, suggesting that elevated hs-CRP levels are associated with an increased risk of frailty [13]. These results are consistent with the findings of this study, suggesting that higher hs-CRP levels are associated with a greater likelihood of frailty. However, another longitudinal survey of older male individuals reported no significant association between CRP and incident frailty based on the Fried phenotype. Such controversial findings may be partly due to differences in the populations studied. Besides, different frailty definitions are plausible explanations, as the Fried phenotype only includes simply physical items, while the FI contains more dimensions.

Of note, previous observational associations are vulnerable to reverse causation biases as frailty may reversely affect the dysregulation of inflammation. MR analyses are less susceptible to residual confounders and reverse causation, which complements the results of observational studies. This is the first time to systematically combine LDSC, pleiotropic research, and MR methods to evaluate the relationship between CRP and frailty. LDSC focuses on estimating heritability and genetic correlation, PLACO aims to identify genetic variants affecting CRP and frailty, and Mendelian randomization investigates causal relationships between CRP and frailty. Based on the large-scale GWAS summary statistics, LDSC was first proposed as a negatively shared genetic overlap between CRP and frailty. Then, PLACO identified 51 shared lead SNPs for the pleiotropic loci. Notably, this study was the first to infer the causal relationship of CRP with frailty risk. We systematically applied the MR analysis to examine the causal relationship of CRP with frailty risk. The causality of CRP-frailty associations was observed using genetic variants associated with CRP as instrument variables. Based on observational and genetic studies, using inflammation as a biomarker for screening middle-aged and older adults at significant risk of frailty could effectively reduce the incidence of frailty and improve the quality of life.

The relationship between inflammation and frailty is complex and multifactorial [7], and the pathophysiological mechanisms need to be better understood. Chronic inflammation may contribute to losing muscle mass, function, and reduced mobility, leading to muscle wasting and weakness. In addition, inflammation can also affect the immune system, leading to immune dysregulation and increased susceptibility to infections. Another proposed mechanism linking inflammation to frailty is the concept of “inflammaging”. This refers to the chronic low-grade inflammation that occurs with ageing, which is thought to be driven by various factors, including cellular damage, oxidative stress, and changes in the gut microbiome. Inflammaging may contribute to the accumulation of cellular damage and dysfunction over time, leading to the development of frailty. Thus, assessment of inflammatory status in older adults may represent a helpful screening test and a potential target for intervention.

In a subgroup analysis based on gender, inflammatory markers hs-CRP predicted progression and regression in the pre-frailty group in both men and women. In other studies, two inflammatory markers at high concentrations in women were better predictors of frailty onset. At the same time, no significant association was observed between inflammatory markers and incident frailty in men [6], which differs from the findings of this study. When participants were divided into middle-aged (45–60 years) and older population (> 60 years), We observed that the relationship between CRP and pre-frailty progression/regression is much more significant among middle-aged people. Interestingly, Cheng et al. found that systemic inflammatory markers were more positively associated with frailty in middle-aged people (45–60 years) compared to older people (> 60 years). This suggested that developing intervention strategies to prevent pre-frailty progression and promote pre-frailty regression may be urgent in the middle-aged population.

### Strengths and limitations

Although we used a comprehensive approach to dissect the relationship between hs-CRP and frailty, some limitations remain. First, this study excluded some subjects for specific criteria, and this non-random selection may lead to selection bias in the results. Second, the total deficit included in the FI calculation is insufficient, which can lead to inaccurate or unstable results. Third, because most of the flaws contained in FI calculations are self-reported, the possibility of information bias cannot be eliminated. In order to better calculate FI, more objective measures of self-reported are needed. Fourth, the demographic makeup in our observational study was entirely middle-aged and older Chinese adults, which may not be fully extrapolated to other populations of all ages or other ethnicities. However, the strength of this study is the combination of a range of genetic approaches, such as LDSC, pleiotropy analysis, and Mr. Methods, to assess the relationship between CRP and frailty. Notably, the study also found for the first time that higher levels of hs-CRP were associated with an increased chance of progression. In contrast, lower hs-CRP levels were associated with an increased probability of regression in pre-debilitated participants.

## Conclusion

Our findings suggest that higher levels of hs-CRP are associated with an increased risk of frailty progression. Besides, we provided strong evidence for the critical role of CRP in the pathogenesis of frailty through genetic approaches. Moreover, pre-frailty individuals with high hs-CRP levels are likelier to progress to frailty and less likely to experience health. However, the underlying mechanism of the inflammatory cytokines leading to frailty is still needed.

### Supplementary Information

**Supplementary Material 1.**

## Data Availability

Data are available and can be downloaded from http://charls.pku.edu.cn/, accessed on 11 February 2023.
