# Supplementary material for: Unraveling the relationship between high-sensitivity C-reactive protein and frailty: evidence from longitudinal cohort study and genetic analysis
Source: BMC Geriatr. 2024 Mar 4;24:222. doi: 10.1186/s12877-024-04836-2 (PMC10913347; doi:10.1186/s12877-024-04836-2)
Supplement: Supplementary file 1 — Supplementary Material 1. [file 12877_2024_4836_MOESM1_ESM.docx]

**Unraveling the Relationship Between High-sensitivity C-reactive Protein and Frailty: Evidence from** **Longitudinal Cohort Study and Genetic Analysis**

**Supplementary files**

**Legend:**

**Table S1** Questionnaire Items of Frailty Index

**Table S2** 297 SNPs as instrumental variable for Mendelian randomization analysis

**Fig.S1** Association between the hs-CRP and frailty according to RSC regression, allowing for nonlinear effects. The ORs and 95 % CI were calculated by multivariate adjustment. Adjusting age, sex, body mass index, marital state, smoke, systolic blood pressure, diastolic blood pressure, drinking status, sleep duration, hypertension, hyperlipidemia, diabetes, and cardiovascular disease.

**Fig.S2** Forest plot of sensitivity analysis of Hs-CRP and frailty; model 1: unadjusted; model 2: adjusted for age, sex; model 3: adjusted for age, sex, body mass index, marital state, smoke, systolic blood pressure, diastolic blood pressure, drinking status, sleep duration, hypertension, hyperlipidemia, diabetes, and cardiovascular disease.

**Fig.S3** Scatter plot of effect of Hs-CRP and frailty in an MR analysis.

**Fig.S4** Funnel plot of the effect of Hs-CRP and frailty in an MR analysis.

**Fig.S5** Leave-one-out plot of the effect of Hs-CRP and frailty in an MR analysis.

**Table S1** Questionnaire Items of Frailty Index

| **Definition** | **Coding of variables** |
| --- | --- |
| Self-reported diagnosis of hypertension by a doctor, self-reported use of antihypertension drugs, systolic blood pressure measured to be ≥ 140mmHg, or Diastolic blood pressure measured to be ≥ 90mmHg | Yes=1.00; no=0.00 |
| Self-reported diagnosis of dyslipidemia by a doctor | Yes=1.00; no=0.00 |
| Self-reported diagnosis of chronic lung diseases by a doctor | Yes=1.00; no=0.00 |
| Self-reported diagnosis of liver diseases by a doctor | Yes=1.00; no=0.00 |
| Self-reported diagnosis of heart attack, coronary heart disease, angina, congestive heart failure, or other heart problems by a doctor | Yes=1.00; no=0.00 |
| Self-reported diagnosis of stroke by a doctor | Yes=1.00; no=0.00 |
| Self-reported diagnosis of stomach or other digestive diseases by a doctor | Yes=1.00; no=0.00 |
| Self-reported diagnosis of emotional, nervous, or psychiatric problems by a doctor | Yes=1.00; no=0.00 |
| Self-reported diagnosis of asthma by a doctor | Yes=1.00; no=0.00 |
| Self-reported diagnosis of memory-related disease by a doctor | Yes=1.00; no=0.00 |
| Self-reported diagnosis of diabetes | Yes=1.00; no=0.00 |
| Self-reported diagnosis of cancer by a doctor | Yes=1.00; no=0.00 |
| Self-reported diagnosis of chronic kidney disease by a doctor | Yes=1.00; no=0.00 |
| Cognitive impairments (MMSE) | <10,1.00; 11-17,0.75; 18-20,0.50;21-24,0.25;>=25,0.00； |
| Depressive symptoms (the CES-D scale) | >=12,1.00; otherwise,0.00 |
| Do you have any difficulty with running or jogging about 1Km? | Yes, I have difficulty and need help, or I can not do it,1.00; otherwise,0.00 |
| Do you have difficulty with walking 1 km? | Yes, I have difficulty and need help, or I can not do it,1.00; otherwise,0.00 |
| Do you have difficulty with walking 100 metres? | Yes, I have difficulty and need help, or I can not do it,1.00; otherwise,0.00 |
| Do you have difficulty with getting up from a chair. After sitting for along period? | Yes, I have difficulty and need help, or I can not do it,1.00; otherwise,0.00 |
| Do you have difficulty with climbing sever alflights of stairs without resting? | Yes, I have difficulty and need help, or I can not do it,1.00; otherwise,0.00 |
| Do you have difficulty with stooping, kneeling, or crouching? | Yes, I have difficulty and need help, or I can not do it,1.00; otherwise,0.00 |
| Do you have difficulty with reaching or extending your arms above shoulder level? | Yes, I have difficulty and need help, or I can not do it,1.00; otherwise,0.00 |
| Do you have difficulty with lifting or carrying weights over10jin? | Yes, I have difficulty and need help, or I can not do it,1.00; otherwise,0.00 |
| Do you have difficulty with picking up a small coin from table? | Yes, I have difficulty and need help, or I can not do it,1.00; otherwise,0.00 |
| Because of health and memory problems, do you have any difficulty with dressing? | Yes, I have difficulty and need help, or I can not do it,1.00; otherwise,0.00 |
| Do you have any difficulty with bathing or showering? | Yes, I have difficulty and need help, or I can not do it,1.00; otherwise,0.00 |
| Do you have any difficulty with eating, such as cutting Up your food? | Yes, I have difficulty and need help, or I can not do it,1.00; otherwise,0.00 |
| Do you have any difficulty with getting into or out of bed? | Yes, I have difficulty and need help, or I can not do it,1.00; otherwise,0.00 |
| Do you have any difficulties with using the toilet, Including getting up and down? | Yes, I have difficulty and need help, or I can not do it,1.00; otherwise,0.00 |
| Do you have any difficulties with controlling urination and defecation? | Yes, I have difficulty and need help, or I can not do it,1.00; otherwise,0.00 |
| Do you have any difficulties with doing household chores? | Yes, I have difficulty and need help, or I can not do it,1.00; otherwise,0.00 |
| Do you have any difficulties with preparing hot meals? | Yes, I have difficulty and need help, or I can not do it,1.00; otherwise,0.00 |
| Do you have any difficulties with shopping for groceries? | Yes, I have difficulty and need help, or I can not do it,1.00; otherwise,0.00 |
| Do you have any difficulties with managing your money | Yes, I have difficulty and need help, or I can not do it,1.00; otherwise,0.00 |
| Do you have any difficulties with taking medications? | Yes, I have difficulty and need help, or I can not do it,1.00; otherwise,0.00 |
| Do you have one of physical or brain damage/intellectual disabilities? | Yes=1.00; no=0.00 |
| Is your hearing very good, good, fair, poor, or very poor? | Very poor,1.00; poor,0.75; fair,0.5; good,0.25; very good,0 |
| How good is your eyesight for seeing things at a distance? | Very poor,1.00; poor,0.75; fair,0.5;good,0.25; very good,0 |
| How good is your eyesight for seeing things up close? | Very poor,1.00; poor,0.75; fair,0.5;good,0.25; very good,0 |
| Would you say your health is very good, good, fair, poor or very poor? | Very poor,1.00; poor,0.75; fair,0.5;good,0.25; very good,0 |
| Think about your life-as-a-whole. How sat is fie dare you with it? | Very poor,1.00; poor,0.75; fair,0.5;good,0.25; very good,0 |

**Table S2 297 SNPs as instrumental variable for Mendelian randomization analysis**

| **SNP** | **effect_allele** | **other_allele** | **beta** | **eaf** | **se** | **pval** | **chr** | **pos** |
| --- | --- | --- | --- | --- | --- | --- | --- | --- |
| rs10169482 | T | G | -0.013864 | 0.3914 | 0.0024528 | 1.58E-08 | 2 | 61486628 |
| rs10203386 | A | T | 0.019282 | 0.45313 | 0.0024021 | 1E-15 | 2 | 25136866 |
| rs1037170 | T | C | 0.027968 | 0.72235 | 0.0026789 | 1.64E-25 | 17 | 72702914 |
| rs10491104 | T | C | 0.014088 | 0.52986 | 0.0023929 | 3.92E-09 | 17 | 15884561 |
| rs10760691 | G | A | 0.017264 | 0.38253 | 0.00253 | 8.9E-12 | 9 | 102281383 |
| rs10769254 | C | G | -0.031326 | 0.19014 | 0.0030679 | 1.79E-24 | 11 | 47362465 |
| rs10794644 | C | G | -0.015502 | 0.68467 | 0.0025773 | 1.8E-09 | 1 | 24470902 |
| rs10810455 | G | C | -0.015096 | 0.44184 | 0.0024091 | 3.7E-10 | 9 | 15866156 |
| rs1081105 | C | A | -0.23375 | 0.027885 | 0.0072539 | 1E-200 | 19 | 45412955 |
| rs10831676 | C | A | -0.014719 | 0.46147 | 0.0023982 | 8.39E-10 | 11 | 11820449 |
| rs10849773 | G | A | -0.082635 | 0.015217 | 0.0098109 | 3.69E-17 | 12 | 121080103 |
| rs10925005 | A | C | -0.017659 | 0.66656 | 0.0025326 | 3.11E-12 | 1 | 247549115 |
| rs11012732 | G | A | 0.014182 | 0.33064 | 0.0025445 | 2.49E-08 | 10 | 21830104 |
| rs11047130 | T | C | 0.016489 | 0.21211 | 0.0029285 | 0.000000018 | 12 | 24006668 |
| rs11065406 | T | C | 0.050762 | 0.48846 | 0.0024876 | 1.67E-92 | 12 | 121490520 |
| rs11145763 | C | A | 0.018209 | 0.43307 | 0.0024099 | 4.16E-14 | 9 | 139263596 |
| rs111514504 | T | C | -0.027474 | 0.11281 | 0.0038159 | 6.04E-13 | 11 | 54796787 |
| rs111690247 | T | C | 0.026559 | 0.090242 | 0.0041664 | 1.84E-10 | 12 | 121885201 |
| rs11227395 | C | A | 0.017753 | 0.23888 | 0.0028148 | 2.85E-10 | 11 | 65829637 |
| rs112608302 | A | G | 0.080065 | 0.016685 | 0.0095728 | 6.1E-17 | 1 | 159582699 |
| rs11264242 | A | C | -0.018092 | 0.47881 | 0.0024157 | 6.95E-14 | 1 | 154663374 |
| rs112689575 | A | G | 0.056201 | 0.020619 | 0.0086331 | 7.53E-11 | 1 | 159550965 |
| rs112695172 | A | G | 0.076913 | 0.020971 | 0.0083608 | 3.62E-20 | 1 | 66163183 |
| rs113004644 | T | C | 0.020613 | 0.15107 | 0.0034145 | 1.57E-09 | 12 | 120372754 |
| rs113439314 | C | T | 0.023535 | 0.10093 | 0.00398 | 3.36E-09 | 19 | 11043158 |
| rs115381557 | T | C | 0.21051 | 0.010334 | 0.011811 | 5.04E-71 | 1 | 159725183 |
| rs11550348 | A | G | -0.0215 | 0.12372 | 0.0036269 | 3.07E-09 | 19 | 46387344 |
| rs11577023 | C | T | -0.016074 | 0.30967 | 0.0025978 | 6.12E-10 | 1 | 222061973 |
| rs11605427 | C | G | -0.019648 | 0.40168 | 0.0024406 | 8.26E-16 | 11 | 59928672 |
| rs116073407 | A | G | 0.062504 | 0.014694 | 0.010079 | 5.59E-10 | 1 | 159650531 |
| rs11617494 | A | G | 0.015725 | 0.24059 | 0.0028114 | 2.23E-08 | 13 | 110383798 |
| rs11627333 | A | C | 0.014802 | 0.26551 | 0.0027092 | 4.67E-08 | 14 | 73213835 |
| rs11657865 | C | A | 0.015186 | 0.61022 | 0.0024787 | 8.98E-10 | 17 | 76387274 |
| rs11666245 | A | G | -0.038461 | 0.046688 | 0.0056591 | 1.07E-11 | 19 | 38229926 |
| rs11668327 | C | G | 0.078096 | 0.17562 | 0.0031761 | 2.2E-133 | 19 | 45398633 |
| rs11669810 | G | T | -0.015844 | 0.63156 | 0.0024857 | 1.84E-10 | 19 | 46871132 |
| rs116805289 | C | A | 0.059501 | 0.023081 | 0.0079531 | 7.37E-14 | 1 | 154510155 |
| rs116902363 | A | G | -0.056769 | 0.020241 | 0.0088566 | 1.46E-10 | 19 | 45246759 |
| rs116971887 | T | G | -0.11442 | 0.045343 | 0.0058138 | 3.5E-86 | 16 | 51170026 |
| rs1169720 | T | C | -0.029968 | 0.32447 | 0.0025507 | 7.25E-32 | 12 | 121641034 |
| rs11708067 | G | A | 0.016296 | 0.24557 | 0.002777 | 4.41E-09 | 3 | 123065778 |
| rs11738559 | T | C | 0.018559 | 0.18914 | 0.0030661 | 1.42E-09 | 5 | 150476129 |
| rs11777625 | C | T | 0.022466 | 0.54572 | 0.0024017 | 8.48E-21 | 8 | 126333642 |
| rs11873305 | C | A | -0.035693 | 0.039067 | 0.0061956 | 8.36E-09 | 18 | 58049192 |
| rs12132412 | G | A | 0.016397 | 0.38815 | 0.0024735 | 3.38E-11 | 1 | 21820042 |
| rs12138486 | T | C | 0.01873 | 0.78161 | 0.0028937 | 9.64E-11 | 1 | 93865391 |
| rs12138629 | A | G | -0.037087 | 0.22388 | 0.0029723 | 1.01E-35 | 1 | 66542345 |
| rs12231235 | A | G | -0.026563 | 0.42655 | 0.0024198 | 4.96E-28 | 12 | 95857690 |
| rs1223801 | A | G | -0.01968 | 0.83655 | 0.0032297 | 1.11E-09 | 1 | 214348141 |
| rs12300845 | T | G | -0.058096 | 0.032763 | 0.0067051 | 4.55E-18 | 12 | 24195798 |
| rs12496226 | T | G | 0.023218 | 0.32056 | 0.0025639 | 1.37E-19 | 3 | 49734040 |
| rs12544798 | G | C | 0.018957 | 0.22285 | 0.0028929 | 5.65E-11 | 8 | 10029599 |
| rs1260326 | C | T | -0.073894 | 0.60695 | 0.002441 | 1E-200 | 2 | 27730940 |
| rs12620844 | C | T | 0.013933 | 0.43086 | 0.0024364 | 1.08E-08 | 2 | 232324510 |
| rs12679106 | T | G | -0.014503 | 0.71068 | 0.0026478 | 4.32E-08 | 8 | 73443198 |
| rs12755229 | G | T | 0.020358 | 0.5328 | 0.0024 | 2.21E-17 | 1 | 65814233 |
| rs12933292 | G | C | -0.019173 | 0.40973 | 0.0024365 | 3.58E-15 | 16 | 69566309 |
| rs12941913 | T | C | 0.015568 | 0.60759 | 0.0024507 | 2.12E-10 | 17 | 1346417 |
| rs12944581 | C | G | 0.030897 | 0.72956 | 0.0028021 | 2.89E-28 | 17 | 76348830 |
| rs12972156 | G | C | -0.20667 | 0.14884 | 0.0033609 | 1E-200 | 19 | 45387459 |
| rs12972784 | T | C | 0.01454 | 0.52716 | 0.00239 | 1.18E-09 | 19 | 44704542 |
| rs12992747 | C | A | -0.016125 | 0.77238 | 0.0028569 | 1.66E-08 | 2 | 178190491 |
| rs12992995 | A | C | -0.015843 | 0.27555 | 0.002681 | 3.44E-09 | 2 | 175197545 |
| rs13013 | A | C | 0.01484 | 0.58384 | 0.0024234 | 9.15E-10 | 10 | 75562161 |
| rs13014038 | C | A | 0.014182 | 0.36371 | 0.0024953 | 1.32E-08 | 2 | 57347944 |
| rs13062093 | G | T | 0.015002 | 0.36575 | 0.002479 | 1.44E-09 | 3 | 35667057 |
| rs13066390 | T | C | 0.014145 | 0.39161 | 0.0024519 | 7.98E-09 | 3 | 137090331 |
| rs13066686 | A | C | -0.01343 | 0.40805 | 0.0024392 | 3.68E-08 | 3 | 94075026 |
| rs13242809 | A | T | -0.026623 | 0.2736 | 0.0026899 | 4.31E-23 | 7 | 22746564 |
| rs1336474 | G | A | -0.017589 | 0.22004 | 0.0028955 | 1.24E-09 | 1 | 65691249 |
| rs1348675 | A | G | 0.019242 | 0.23265 | 0.002873 | 2.12E-11 | 13 | 58712638 |
| rs1353792 | C | A | 0.017549 | 0.2393 | 0.0028062 | 4.02E-10 | 12 | 24180307 |
| rs1386821 | G | T | -0.059833 | 0.20506 | 0.0029523 | 2.87E-91 | 1 | 154382049 |
| rs138692741 | T | C | -0.041249 | 0.035547 | 0.0066376 | 5.16E-10 | 19 | 45467132 |
| rs1408272 | G | T | 0.031054 | 0.079856 | 0.0044063 | 1.82E-12 | 6 | 25842951 |
| rs141094656 | C | T | 0.060537 | 0.014992 | 0.01028 | 3.89E-09 | 1 | 92753336 |
| rs141179989 | T | C | -0.05519 | 0.017022 | 0.0099727 | 3.13E-08 | 16 | 51067084 |
| rs141651106 | A | G | 0.043971 | 0.029168 | 0.0072422 | 1.27E-09 | 16 | 51223317 |
| rs143345163 | C | A | 0.062578 | 0.018434 | 0.0091187 | 6.77E-12 | 12 | 121453258 |
| rs143674704 | G | A | -0.074184 | 0.017955 | 0.0093817 | 2.64E-15 | 19 | 45458466 |
| rs143699354 | T | C | -0.066561 | 0.011354 | 0.011909 | 2.29E-08 | 1 | 66297805 |
| rs1441171 | T | G | -0.022503 | 0.52374 | 0.0023924 | 5.17E-21 | 2 | 214033637 |
| rs144970957 | C | T | -0.11011 | 0.021749 | 0.0083638 | 1.42E-39 | 1 | 159514964 |
| rs145147679 | A | G | 0.061912 | 0.017049 | 0.0099871 | 5.68E-10 | 1 | 159787423 |
| rs145244672 | G | T | 0.041466 | 0.12306 | 0.0040374 | 9.65E-25 | 6 | 32556461 |
| rs145643790 | C | T | -0.039427 | 0.040308 | 0.0062563 | 2.94E-10 | 2 | 27231983 |
| rs146297275 | C | G | 0.2197 | 0.013449 | 0.010531 | 1.35E-96 | 1 | 159703857 |
| rs146588776 | C | G | -0.057568 | 0.020708 | 0.0085552 | 1.71E-11 | 1 | 154050245 |
| rs146861677 | A | C | 0.069128 | 0.029399 | 0.0073395 | 4.6E-21 | 12 | 120993415 |
| rs1476698 | G | A | 0.017568 | 0.36912 | 0.0024754 | 1.28E-12 | 2 | 242296449 |
| rs1490384 | T | C | -0.030927 | 0.4982 | 0.00239 | 2.73E-38 | 6 | 126851160 |
| rs149624078 | T | C | -0.12997 | 0.013969 | 0.01045 | 1.69E-35 | 15 | 53728710 |
| rs150310653 | C | G | 0.12197 | 0.0095401 | 0.012594 | 3.53E-22 | 1 | 159720783 |
| rs150844304 | C | A | 0.080146 | 0.024558 | 0.0077278 | 3.38E-25 | 15 | 43726625 |
| rs1532085 | G | A | -0.016115 | 0.61333 | 0.0024532 | 5.07E-11 | 15 | 58683366 |
| rs1545536 | T | C | -0.019732 | 0.21981 | 0.0028854 | 8.01E-12 | 8 | 144643169 |
| rs1566085 | T | G | -0.014228 | 0.54584 | 0.0024174 | 3.97E-09 | 8 | 142624527 |
| rs1653608 | A | G | -0.050784 | 0.020332 | 0.0084751 | 2.07E-09 | 12 | 121706733 |
| rs16835819 | C | T | -0.077323 | 0.018318 | 0.0089868 | 7.73E-18 | 1 | 154108501 |
| rs17051926 | T | C | -0.034171 | 0.055297 | 0.0052258 | 6.21E-11 | 5 | 156735380 |
| rs17138478 | A | C | 0.034379 | 0.12887 | 0.0035674 | 5.62E-22 | 17 | 36073320 |
| rs172305 | A | G | -0.014179 | 0.31307 | 0.0025805 | 3.91E-08 | 5 | 107316915 |
| rs1727748 | T | C | -0.020461 | 0.84225 | 0.0033108 | 6.41E-10 | 19 | 45048323 |
| rs17308476 | T | C | 0.022016 | 0.11129 | 0.0038034 | 7.11E-09 | 2 | 174868755 |
| rs17539995 | A | G | 0.018173 | 0.17823 | 0.003168 | 9.68E-09 | 9 | 92241847 |
| rs17616063 | G | A | -0.12572 | 0.076034 | 0.0045028 | 2.33E-171 | 16 | 51436882 |
| rs17626434 | G | C | -0.01541 | 0.3324 | 0.0025424 | 1.35E-09 | 2 | 114041976 |
| rs1800693 | C | T | -0.019651 | 0.40142 | 0.0024353 | 7.1E-16 | 12 | 6440009 |
| rs1800961 | T | C | -0.1052 | 0.03141 | 0.0068458 | 2.86E-53 | 20 | 43042364 |
| rs1800973 | A | C | 0.02996 | 0.061166 | 0.0049843 | 1.85E-09 | 12 | 69744014 |
| rs180689986 | A | G | 0.21908 | 0.01442 | 0.010372 | 5.69E-99 | 1 | 159701152 |
| rs1811472 | C | G | -0.1197 | 0.4044 | 0.0024545 | 1E-200 | 1 | 159642349 |
| rs185320691 | C | G | 0.041267 | 0.1066 | 0.0042849 | 5.96E-22 | 6 | 32490292 |
| rs186730517 | A | G | 0.073025 | 0.01256 | 0.011872 | 7.72E-10 | 12 | 121682161 |
| rs1883711 | C | G | 0.046048 | 0.031063 | 0.0070171 | 5.31E-11 | 20 | 39179822 |
| rs1905505 | A | G | 0.01947 | 0.28358 | 0.0026507 | 2.06E-13 | 3 | 170695426 |
| rs1933736 | C | T | 0.019016 | 0.40065 | 0.0024385 | 6.28E-15 | 6 | 116387255 |
| rs1979377 | C | A | 0.02616 | 0.069349 | 0.0047676 | 4.09E-08 | 19 | 45259002 |
| rs1985157 | C | T | 0.016339 | 0.41293 | 0.0024272 | 1.68E-11 | 19 | 18513594 |
| rs2011689 | G | A | -0.013459 | 0.6106 | 0.0024522 | 4.06E-08 | 8 | 64341554 |
| rs2017861 | A | G | 0.016593 | 0.27843 | 0.0026794 | 5.92E-10 | 11 | 61481928 |
| rs2030291 | T | A | -0.014378 | 0.38781 | 0.002454 | 4.66E-09 | 11 | 16251251 |
| rs204523 | T | A | -0.017778 | 0.80993 | 0.003062 | 6.4E-09 | 19 | 44958980 |
| rs2049045 | C | G | -0.022944 | 0.18684 | 0.0030643 | 7.03E-14 | 11 | 27694241 |
| rs204914 | T | C | 0.05883 | 0.046788 | 0.0056675 | 3.07E-25 | 19 | 45466335 |
| rs2050392 | A | G | -0.016394 | 0.61192 | 0.0024845 | 4.16E-11 | 10 | 30691503 |
| rs2068888 | A | G | -0.016819 | 0.44895 | 0.0023997 | 2.41E-12 | 10 | 94839642 |
| rs2071190 | A | T | 0.049028 | 0.25461 | 0.0027473 | 3.33E-71 | 12 | 121431272 |
| rs2110944 | C | T | 0.015728 | 0.53073 | 0.0024027 | 5.91E-11 | 2 | 37090233 |
| rs2161037 | A | G | 0.021768 | 0.54527 | 0.0024193 | 2.32E-19 | 2 | 169893419 |
| rs2161374 | T | C | -0.016553 | 0.48653 | 0.0023894 | 4.28E-12 | 5 | 172176886 |
| rs2166625 | C | G | -0.014387 | 0.37187 | 0.0024714 | 5.84E-09 | 13 | 42584871 |
| rs2169385 | A | G | 0.044052 | 0.86354 | 0.0034863 | 1.37E-36 | 8 | 9206678 |
| rs2172131 | C | T | -0.013338 | 0.58031 | 0.002422 | 3.66E-08 | 10 | 133978962 |
| rs2178464 | C | T | -0.090746 | 0.02492 | 0.0076942 | 4.25E-32 | 12 | 121441440 |
| rs2239198 | C | T | 0.028902 | 0.50869 | 0.0023971 | 1.81E-33 | 12 | 120796408 |
| rs2239222 | G | A | 0.034274 | 0.34858 | 0.0025222 | 4.78E-42 | 14 | 73011885 |
| rs2246833 | T | C | 0.026285 | 0.34047 | 0.0025178 | 1.65E-25 | 10 | 91005854 |
| rs2250010 | T | C | 0.02225 | 0.81022 | 0.0030446 | 2.72E-13 | 12 | 47193818 |
| rs2403116 | G | A | 0.013427 | 0.57706 | 0.0024268 | 3.15E-08 | 12 | 84110605 |
| rs2432195 | T | C | 0.01812 | 0.82669 | 0.0031588 | 9.68E-09 | 5 | 56120413 |
| rs2472188 | G | C | 0.021972 | 0.60303 | 0.0024427 | 2.37E-19 | 2 | 113820814 |
| rs2607013 | T | C | 0.026131 | 0.18151 | 0.0031193 | 5.44E-17 | 6 | 31820400 |
| rs2668243 | C | T | 0.058186 | 0.969365 | 0.0072753 | 1.27E-15 | 12 | 121755276 |
| rs2700938 | C | T | 0.022097 | 0.37635 | 0.0024707 | 3.78E-19 | 7 | 36085142 |
| rs2740479 | A | G | 0.016034 | 0.67839 | 0.0025666 | 4.19E-10 | 9 | 107563437 |
| rs2759649 | T | A | 0.014485 | 0.39214 | 0.0024502 | 3.39E-09 | 1 | 198779991 |
| rs2777895 | G | A | 0.019221 | 0.55108 | 0.0023977 | 1.09E-15 | 17 | 57879610 |
| rs2836881 | T | G | -0.03173 | 0.26662 | 0.0027048 | 8.98E-32 | 21 | 40466299 |
| rs2847289 | C | A | 0.022248 | 0.57246 | 0.0024163 | 3.36E-20 | 18 | 12812167 |
| rs28601761 | G | C | -0.022717 | 0.41938 | 0.0024499 | 1.83E-20 | 8 | 126500031 |
| rs28929474 | T | C | -0.10043 | 0.020304 | 0.0084753 | 2.19E-32 | 14 | 94844947 |
| rs2927434 | C | T | 0.032998 | 0.80409 | 0.0030156 | 7.31E-28 | 19 | 45236729 |
| rs2961111 | T | C | 0.020958 | 0.16488 | 0.0032238 | 7.98E-11 | 8 | 103506057 |
| rs2965156 | C | G | 0.021754 | 0.51578 | 0.0023887 | 8.5E-20 | 19 | 45188429 |
| rs2975720 | G | A | 0.02184 | 0.10961 | 0.0038308 | 1.19E-08 | 8 | 10097511 |
| rs2997468 | C | G | -0.016307 | 0.36044 | 0.002524 | 1.04E-10 | 10 | 81073763 |
| rs3094508 | C | T | -0.014463 | 0.41002 | 0.0024454 | 3.33E-09 | 17 | 36062935 |
| rs3125326 | C | A | 0.014007 | 0.60889 | 0.0024712 | 1.45E-08 | 10 | 63053788 |
| rs340005 | A | G | 0.030559 | 0.62058 | 0.0024597 | 1.97E-35 | 15 | 60878030 |
| rs34139656 | G | A | 0.019717 | 0.3277 | 0.0025614 | 1.39E-14 | 16 | 88534923 |
| rs34284056 | A | C | -0.015149 | 0.27697 | 0.002677 | 1.52E-08 | 18 | 60203855 |
| rs34298354 | T | C | -0.026962 | 0.12167 | 0.0036678 | 1.97E-13 | 1 | 247588053 |
| rs34298980 | C | T | -0.014548 | 0.50726 | 0.0025196 | 7.75E-09 | 6 | 40409243 |
| rs34761529 | T | C | -0.016628 | 0.20477 | 0.0029717 | 0.000000022 | 1 | 22681214 |
| rs34875040 | G | A | 0.023914 | 0.13194 | 0.0035309 | 1.27E-11 | 1 | 66265834 |
| rs34982954 | C | T | -0.013657 | 0.47492 | 0.0023918 | 1.13E-08 | 16 | 79040870 |
| rs35371668 | T | C | 0.04064 | 0.18084 | 0.003156 | 6.19E-38 | 6 | 32561638 |
| rs35635959 | C | T | 0.019093 | 0.28661 | 0.0026424 | 5E-13 | 17 | 40772288 |
| rs35764600 | C | G | -0.013689 | 0.39802 | 0.0024702 | 2.99E-08 | 12 | 11791628 |
| rs35881303 | G | A | -0.014609 | 0.57907 | 0.0024236 | 1.67E-09 | 6 | 33461779 |
| rs36057734 | C | T | 0.016796 | 0.26862 | 0.0027093 | 5.67E-10 | 17 | 68394967 |
| rs3760091 | G | C | -0.015405 | 0.39472 | 0.0025463 | 1.45E-09 | 16 | 28620800 |
| rs3768321 | T | G | 0.032417 | 0.1967 | 0.0030107 | 4.96E-27 | 1 | 40035928 |
| rs385417 | C | A | -0.015998 | 0.61683 | 0.0024624 | 8.21E-11 | 7 | 101864836 |
| rs3865444 | A | C | -0.018972 | 0.32555 | 0.0025487 | 9.81E-14 | 19 | 51727962 |
| rs3935032 | T | C | -0.018415 | 0.37726 | 0.0025052 | 1.98E-13 | 1 | 1564194 |
| rs4006577 | A | G | -0.014357 | 0.62097 | 0.0024742 | 6.54E-09 | 1 | 236301301 |
| rs4074793 | G | A | 0.026618 | 0.07444 | 0.0045549 | 5.11E-09 | 5 | 52193125 |
| rs4148155 | G | A | -0.021269 | 0.1144 | 0.0037506 | 1.42E-08 | 4 | 89054667 |
| rs41523449 | A | G | -0.022721 | 0.16513 | 0.0033612 | 1.38E-11 | 19 | 35537706 |
| rs4404788 | A | G | 0.022546 | 0.67328 | 0.0025431 | 7.65E-19 | 6 | 130369323 |
| rs4426089 | T | C | -0.013101 | 0.45473 | 0.0024014 | 4.89E-08 | 10 | 62223112 |
| rs4609871 | T | C | 0.016003 | 0.55151 | 0.0024013 | 2.66E-11 | 16 | 29932064 |
| rs4655537 | G | A | -0.10079 | 0.62857 | 0.0024741 | 1E-200 | 1 | 66058801 |
| rs469802 | G | A | -0.03502 | 0.20398 | 0.0029884 | 1.04E-31 | 1 | 91545826 |
| rs4704093 | G | T | 0.017069 | 0.46801 | 0.0024048 | 1.27E-12 | 5 | 72976175 |
| rs4714508 | G | A | 0.018294 | 0.34491 | 0.0025282 | 4.63E-13 | 6 | 41671677 |
| rs4764939 | T | C | -0.017859 | 0.46947 | 0.0023971 | 9.33E-14 | 12 | 103522952 |
| rs4767921 | G | A | -0.035994 | 0.63119 | 0.0024852 | 1.6E-47 | 12 | 121069201 |
| rs4790286 | A | T | -0.019557 | 0.21875 | 0.0028894 | 1.3E-11 | 17 | 1652483 |
| rs4802070 | G | A | -0.015051 | 0.73535 | 0.0027103 | 2.81E-08 | 19 | 40726336 |
| rs4811031 | G | A | 0.017805 | 0.34691 | 0.0025114 | 1.35E-12 | 20 | 48956954 |
| rs4851487 | T | C | 0.015483 | 0.39709 | 0.0024468 | 2.49E-10 | 2 | 102302978 |
| rs4871827 | A | G | 0.014209 | 0.32599 | 0.0025476 | 2.44E-08 | 8 | 121061879 |
| rs4925671 | C | T | 0.018792 | 0.69744 | 0.0025997 | 4.9E-13 | 1 | 247622874 |
| rs493098 | G | C | -0.014931 | 0.42597 | 0.0024165 | 6.46E-10 | 1 | 66671953 |
| rs4970834 | T | C | 0.019464 | 0.18769 | 0.003075 | 2.46E-10 | 1 | 109814880 |
| rs519790 | G | C | 0.018633 | 0.34157 | 0.0025172 | 1.34E-13 | 11 | 72504141 |
| rs55855238 | C | T | 0.026122 | 0.65051 | 0.0025055 | 1.91E-25 | 18 | 55089715 |
| rs55981844 | C | T | -0.019309 | 0.29979 | 0.0026105 | 1.4E-13 | 14 | 96933414 |
| rs56015600 | G | A | 0.039944 | 0.6297 | 0.0024753 | 1.48E-58 | 1 | 247601886 |
| rs56094641 | G | A | 0.021778 | 0.40295 | 0.0024375 | 4.12E-19 | 16 | 53806453 |
| rs567910619 | T | C | -0.088795 | 0.010854 | 0.012167 | 2.93E-13 | 1 | 65210790 |
| rs583515 | T | C | -0.013158 | 0.48618 | 0.0023899 | 3.68E-08 | 1 | 10492684 |
| rs601338 | A | G | 0.024612 | 0.51058 | 0.0023984 | 1.06E-24 | 19 | 49206674 |
| rs6073958 | C | T | -0.028748 | 0.19847 | 0.0029961 | 8.42E-22 | 20 | 44551855 |
| rs60821336 | C | T | 0.056581 | 0.02221 | 0.0081184 | 3.18E-12 | 16 | 51398163 |
| rs6083801 | T | C | -0.015637 | 0.52661 | 0.0023942 | 6.53E-11 | 20 | 25307574 |
| rs6089985 | T | C | -0.015025 | 0.34133 | 0.0027087 | 2.91E-08 | 20 | 61377272 |
| rs61542988 | T | C | -0.026486 | 0.24604 | 0.0027863 | 1.99E-21 | 7 | 22882291 |
| rs61806853 | C | T | -0.043505 | 0.049831 | 0.0054886 | 2.26E-15 | 1 | 154154587 |
| rs62011286 | A | G | -0.015791 | 0.34198 | 0.0025189 | 3.63E-10 | 15 | 63791125 |
| rs62104180 | A | G | -0.032276 | 0.050106 | 0.00548 | 3.87E-09 | 2 | 466003 |
| rs62129471 | A | G | 0.014007 | 0.65802 | 0.0025673 | 4.87E-08 | 19 | 1950864 |
| rs62282070 | C | T | -0.026395 | 0.092123 | 0.0041429 | 1.88E-10 | 3 | 141888148 |
| rs62493588 | A | C | 0.021441 | 0.12542 | 0.0037266 | 8.75E-09 | 8 | 11774411 |
| rs62513181 | G | A | 0.028595 | 0.065321 | 0.0048212 | 3.01E-09 | 8 | 129126459 |
| rs62618693 | T | C | -0.031471 | 0.045369 | 0.0057285 | 3.94E-08 | 11 | 32956492 |
| rs635634 | T | C | 0.032827 | 0.18392 | 0.0030985 | 3.19E-26 | 9 | 136155000 |
| rs6486122 | T | C | 0.028659 | 0.69114 | 0.0025831 | 1.34E-28 | 11 | 13361524 |
| rs6501207 | C | T | -0.031294 | 0.73239 | 0.0027648 | 1.07E-29 | 17 | 76364099 |
| rs6519133 | C | T | -0.033229 | 0.39346 | 0.0024477 | 5.73E-42 | 22 | 39096602 |
| rs653170 | T | C | 0.015591 | 0.35929 | 0.0024922 | 3.96E-10 | 1 | 112328245 |
| rs66744209 | G | A | 0.024758 | 0.21438 | 0.0029289 | 2.85E-17 | 16 | 51123320 |
| rs6677719 | T | C | 0.051783 | 0.6475 | 0.0025097 | 1.58E-94 | 1 | 159723120 |
| rs6698653 | C | T | 0.06321 | 0.47739 | 0.0023895 | 4.75E-154 | 1 | 65944850 |
| rs6734238 | G | A | 0.043305 | 0.40205 | 0.002433 | 7.74E-71 | 2 | 113841030 |
| rs67581262 | G | C | 0.013341 | 0.47982 | 0.002397 | 2.61E-08 | 3 | 196045555 |
| rs67881993 | T | G | -0.043171 | 0.052781 | 0.0054046 | 1.38E-15 | 12 | 121565950 |
| rs67919109 | T | G | 0.023886 | 0.10749 | 0.0038575 | 5.94E-10 | 1 | 198147819 |
| rs6792725 | G | A | -0.017916 | 0.69345 | 0.0026702 | 1.96E-11 | 3 | 24520283 |
| rs6845703 | A | G | 0.013937 | 0.66233 | 0.0025274 | 0.000000035 | 4 | 45134713 |
| rs687339 | T | C | -0.026276 | 0.77192 | 0.0028508 | 3.07E-20 | 3 | 135932359 |
| rs6905544 | G | A | 0.018064 | 0.59988 | 0.0024455 | 1.51E-13 | 6 | 98411631 |
| rs6910879 | G | A | 0.040548 | 0.092176 | 0.0043434 | 1.01E-20 | 6 | 32560739 |
| rs6920220 | A | G | 0.021333 | 0.22253 | 0.0028621 | 9.11E-14 | 6 | 138006504 |
| rs6961634 | A | G | -0.02708 | 0.14946 | 0.0033539 | 6.82E-16 | 7 | 99181839 |
| rs6962836 | G | A | 0.019583 | 0.1393 | 0.0034945 | 0.000000021 | 7 | 22705474 |
| rs7012637 | A | G | 0.047506 | 0.47495 | 0.0024076 | 1.29E-86 | 8 | 9173209 |
| rs704017 | G | A | -0.01859 | 0.57174 | 0.0024143 | 1.36E-14 | 10 | 80819132 |
| rs7084062 | G | A | 0.018021 | 0.48874 | 0.0024165 | 8.85E-14 | 10 | 133736636 |
| rs714052 | G | A | -0.031432 | 0.12458 | 0.0036136 | 3.38E-18 | 7 | 72864869 |
| rs7208261 | T | G | 0.016105 | 0.71332 | 0.0026384 | 1.04E-09 | 17 | 73156068 |
| rs72636674 | C | G | 0.018618 | 0.166 | 0.0032155 | 7.04E-09 | 4 | 67998903 |
| rs728455 | T | C | 0.014075 | 0.38575 | 0.0024715 | 1.24E-08 | 2 | 172034785 |
| rs73001065 | C | G | 0.042886 | 0.070962 | 0.0046672 | 3.99E-20 | 19 | 19460541 |
| rs73020724 | C | A | -0.070428 | 0.01901 | 0.0088767 | 2.13E-15 | 1 | 159581435 |
| rs7311631 | G | A | -0.016481 | 0.49281 | 0.002387 | 5.05E-12 | 12 | 90429028 |
| rs73137144 | G | A | -0.018512 | 0.19289 | 0.0030283 | 9.8E-10 | 7 | 74073590 |
| rs7317323 | C | T | 0.029144 | 0.057036 | 0.0051496 | 1.52E-08 | 13 | 42957489 |
| rs7357754 | G | A | 0.020787 | 0.49999 | 0.0023961 | 4.14E-18 | 9 | 92207308 |
| rs7442885 | G | C | -0.016244 | 0.21007 | 0.0029423 | 3.38E-08 | 5 | 87682877 |
| rs74590348 | G | A | -0.055923 | 0.016599 | 0.0095063 | 4.04E-09 | 1 | 153823812 |
| rs74607435 | C | T | 0.042952 | 0.051226 | 0.0054194 | 2.28E-15 | 19 | 45235700 |
| rs7488791 | A | G | -0.013143 | 0.54134 | 0.0024087 | 4.87E-08 | 12 | 125334068 |
| rs7500375 | A | C | -0.032515 | 0.64286 | 0.0025103 | 2.31E-38 | 16 | 51194301 |
| rs75031836 | A | C | 0.051872 | 0.016859 | 0.00947 | 4.32E-08 | 2 | 28487135 |
| rs75064168 | A | G | 0.024337 | 0.091035 | 0.0041883 | 6.22E-09 | 3 | 101845939 |
| rs75178253 | G | A | 0.056511 | 0.078301 | 0.0047296 | 6.72E-33 | 19 | 45461007 |
| rs7537072 | C | T | -0.01337 | 0.41631 | 0.0024304 | 3.78E-08 | 1 | 21434533 |
| rs75460349 | C | A | -0.091093 | 0.023662 | 0.0079836 | 3.77E-30 | 1 | 27180088 |
| rs7563362 | G | A | 0.02111 | 0.85773 | 0.0034353 | 8.01E-10 | 2 | 620297 |
| rs7599739 | T | G | -0.016966 | 0.34968 | 0.0025063 | 1.29E-11 | 2 | 27363152 |
| rs76040012 | C | T | -0.023351 | 0.10177 | 0.0039801 | 4.44E-09 | 2 | 59150981 |
| rs76448484 | C | T | 0.037691 | 0.054649 | 0.005313 | 1.3E-12 | 8 | 9155582 |
| rs7652415 | T | C | 0.023481 | 0.12802 | 0.0035763 | 5.19E-11 | 3 | 9505238 |
| rs76819459 | G | A | 0.017095 | 0.32698 | 0.0025485 | 1.98E-11 | 16 | 79718917 |
| rs76870047 | G | A | 0.056447 | 0.043857 | 0.006043 | 9.6E-21 | 1 | 65281910 |
| rs76934186 | C | T | -0.042811 | 0.028056 | 0.0073717 | 6.35E-09 | 1 | 66250890 |
| rs76962533 | T | C | -0.063522 | 0.038133 | 0.0062371 | 2.34E-24 | 1 | 66194165 |
| rs77509028 | T | G | 0.06519 | 0.015565 | 0.009638 | 1.35E-11 | 1 | 159740174 |
| rs77704739 | C | T | -0.051242 | 0.042351 | 0.0059437 | 6.65E-18 | 5 | 52080909 |
| rs7808907 | C | T | 0.013756 | 0.50057 | 0.002389 | 8.52E-09 | 7 | 128584084 |
| rs78273125 | T | A | -0.038932 | 0.092296 | 0.0041261 | 3.91E-21 | 19 | 45644354 |
| rs7828742 | G | A | 0.026336 | 0.5981 | 0.0024497 | 5.94E-27 | 8 | 116960729 |
| rs7833554 | T | C | 0.013932 | 0.555 | 0.0024083 | 7.26E-09 | 8 | 103642986 |
| rs78343493 | A | G | 0.021749 | 0.11938 | 0.0036915 | 3.83E-09 | 2 | 165502911 |
| rs78620885 | T | C | -0.058788 | 0.030478 | 0.0069535 | 2.82E-17 | 19 | 45591084 |
| rs79101008 | A | C | -0.029742 | 0.11065 | 0.0038068 | 5.61E-15 | 15 | 53076370 |
| rs79429216 | A | G | -0.086826 | 0.012389 | 0.010779 | 7.96E-16 | 19 | 45445517 |
| rs7956514 | G | T | 0.016997 | 0.28665 | 0.0026374 | 1.16E-10 | 12 | 12879254 |
| rs79875796 | C | A | 0.049893 | 0.037347 | 0.0064098 | 7.05E-15 | 12 | 121699887 |
| rs799260 | A | G | -0.022898 | 0.16622 | 0.0032052 | 9.08E-13 | 12 | 56921304 |
| rs80257887 | A | G | -0.046577 | 0.036064 | 0.0064059 | 3.58E-13 | 19 | 45020859 |
| rs80292319 | C | T | -0.028166 | 0.058721 | 0.0050841 | 3.03E-08 | 15 | 76508632 |
| rs8060025 | G | T | -0.01854 | 0.61106 | 0.0024505 | 3.87E-14 | 16 | 27327214 |
| rs8110504 | G | A | 0.019086 | 0.84583 | 0.0033561 | 1.29E-08 | 19 | 47046668 |
| rs8126001 | T | C | -0.015684 | 0.48971 | 0.0023975 | 6.09E-11 | 20 | 62711459 |
| rs8178824 | T | C | 0.060272 | 0.029741 | 0.0070548 | 1.31E-17 | 17 | 64224775 |
| rs863013 | T | G | -0.044276 | 0.34111 | 0.0025234 | 6.76E-69 | 1 | 159200020 |
| rs879620 | T | C | 0.013732 | 0.61544 | 0.0024666 | 2.59E-08 | 16 | 4015729 |
| rs9366639 | G | C | -0.029243 | 0.17839 | 0.0031156 | 6.27E-21 | 6 | 26167613 |
| rs9604045 | T | G | -0.022968 | 0.25026 | 0.0028746 | 1.36E-15 | 13 | 113927208 |
| rs9611454 | T | C | -0.018054 | 0.35206 | 0.002517 | 7.36E-13 | 22 | 41409429 |
| rs9638882 | A | C | -0.024383 | 0.20879 | 0.0029448 | 1.24E-16 | 7 | 1023617 |
| rs9738365 | A | C | 0.02034 | 0.26663 | 0.0027085 | 5.94E-14 | 12 | 31997635 |


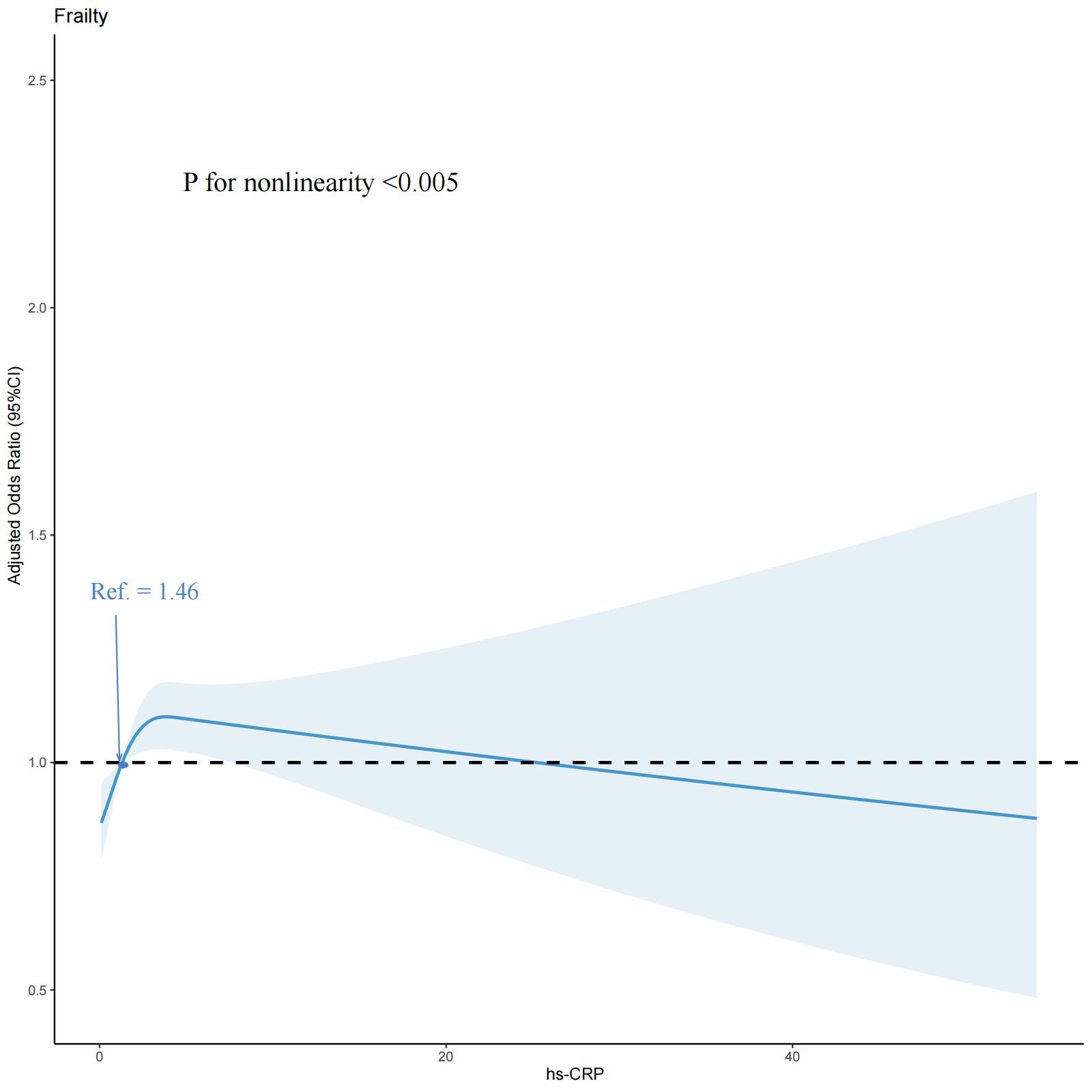


**Fig.S1** Association between the hs-CRP and frailty according to RSC regression, allowing for nonlinear effects. The ORs and 95 % CI were calculated by multivariate adjustment. Adjusting age, sex, body mass index, marital state, smoke, systolic blood pressure, diastolic blood pressure, drinking status, sleep duration, hypertension, hyperlipidemia, diabetes, and cardiovascular disease.


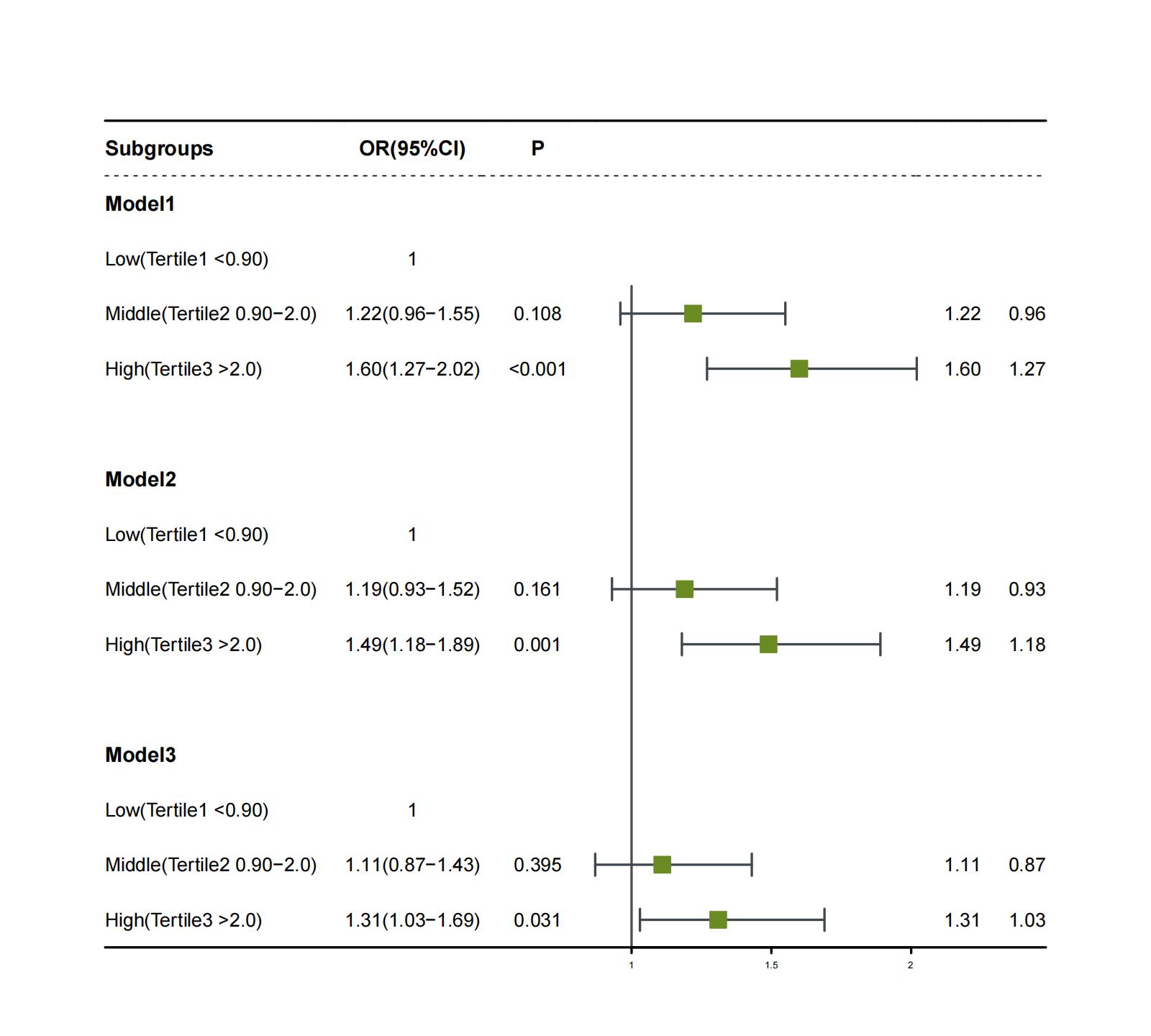


**Fig.S2** Forest plot of sensitivity analysis of Hs-CRP and frailty; model 1: unadjusted; model 2: adjusted for age, sex; model 3: adjusted for age, sex, body mass index, marital state, smoke, systolic blood pressure, diastolic blood pressure, drinking status, sleep duration, hypertension, hyperlipidemia, diabetes, and cardiovascular disease.


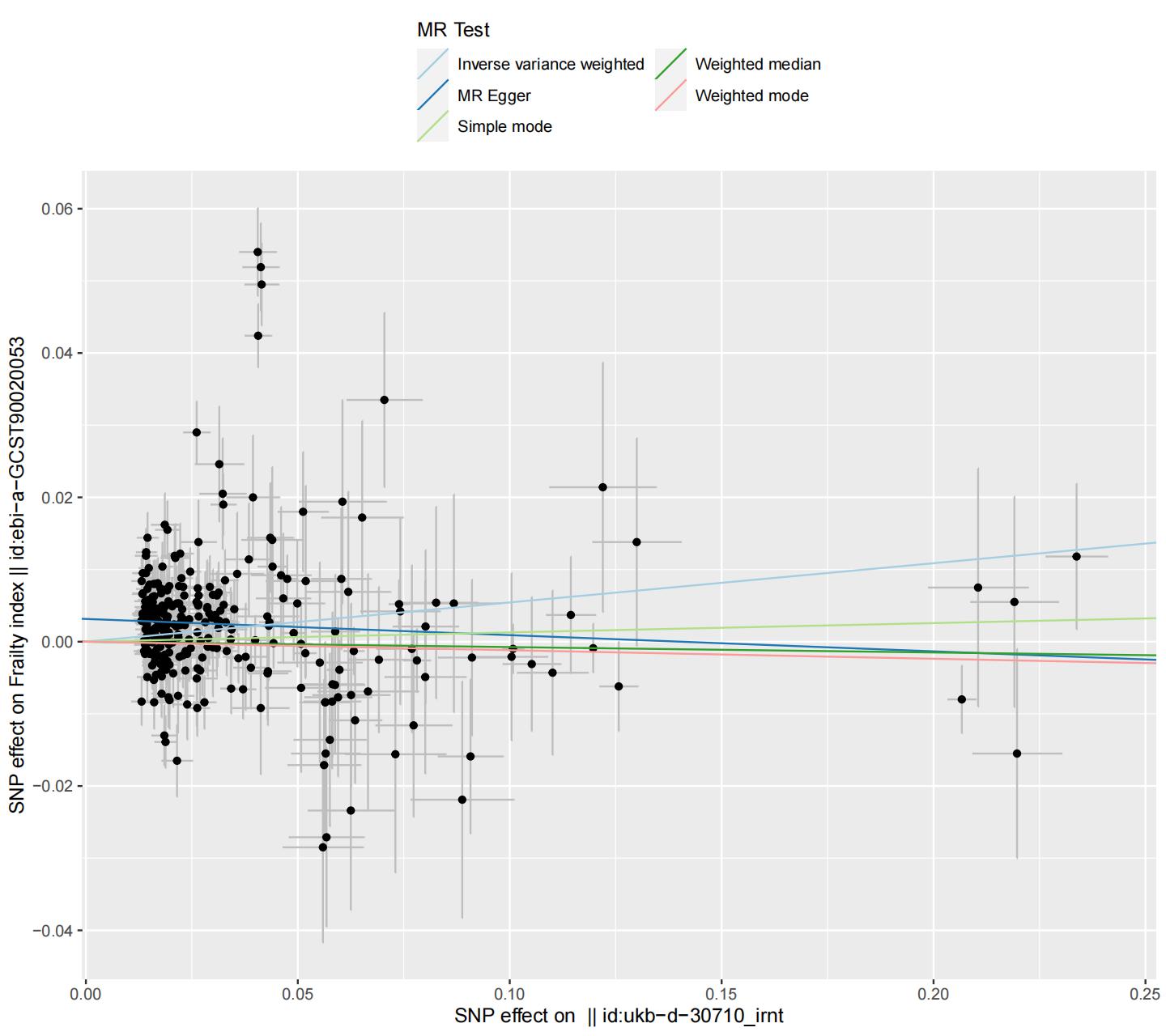


**Fig.S3** Scatter plot of effect of Hs-CRP and frailty in an MR analysis.


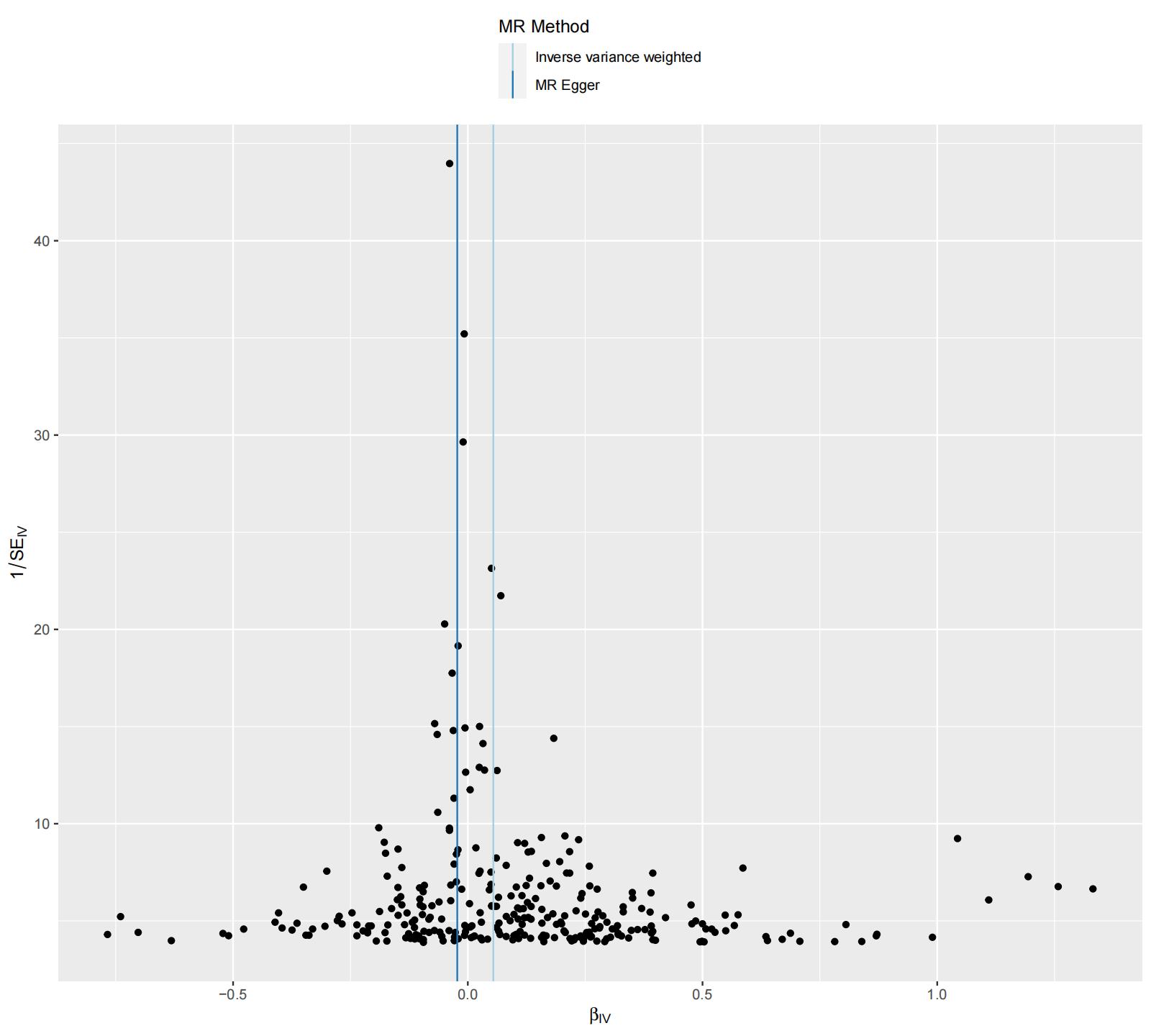


**Fig.S4** Funnel plot of the effect of Hs-CRP and frailty in an MR analysis.


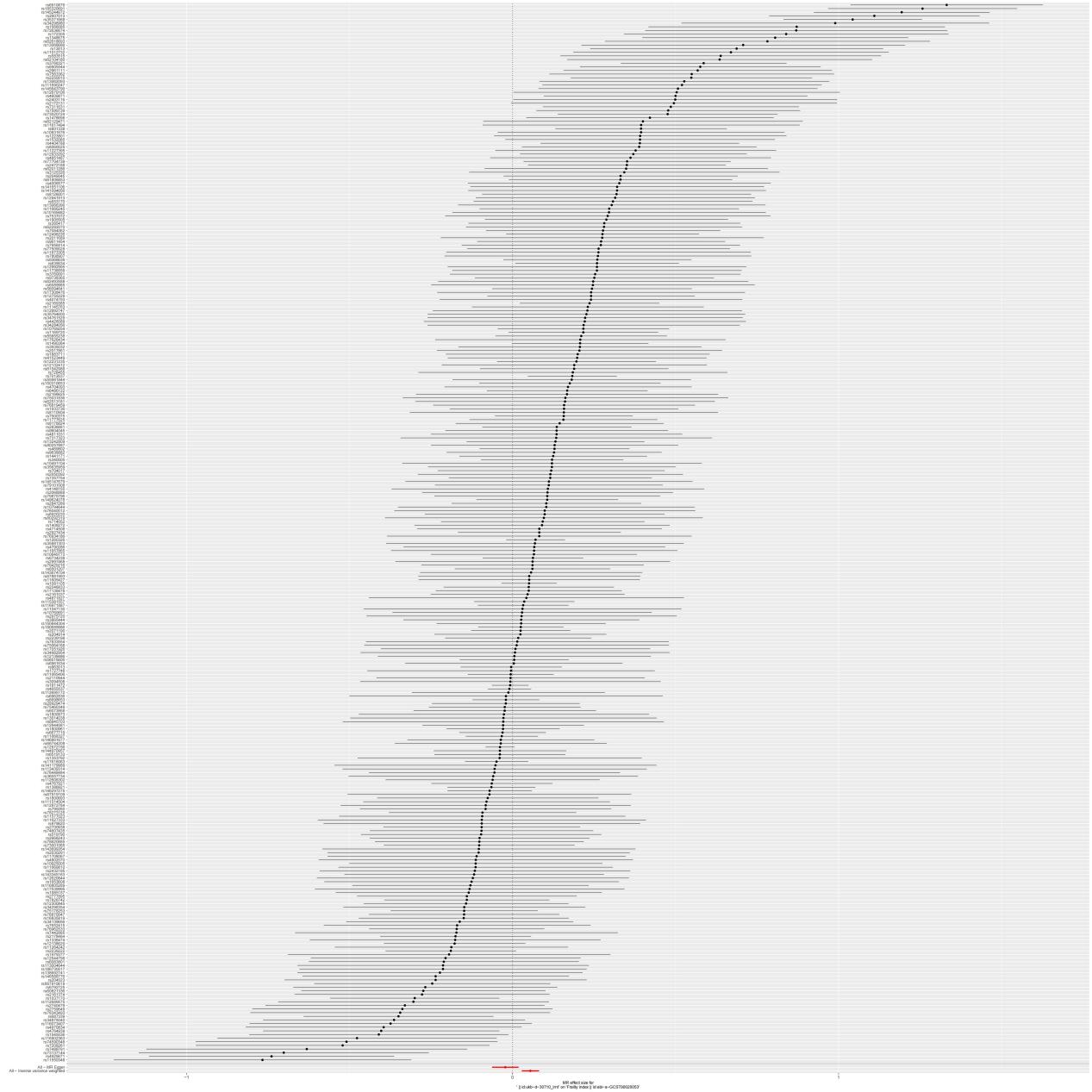


**Fig.S5** Leave-one-out plot of the effect of Hs-CRP and frailty in an MR analysis.
